# What is the impact on health and wellbeing of interventions that foster respect and social inclusion in community-residing older adults? A systematic review of quantitative and qualitative studies

**DOI:** 10.1186/s13643-018-0680-2

**Published:** 2018-01-30

**Authors:** S. Ronzi, L. Orton, D. Pope, N. K. Valtorta, N. G. Bruce

**Affiliations:** 1 Department of Public Health and Policy, University of Liverpool, Liverpool, UK; 2 Institute for Ageing, Newcastle University, Newcastle upon Tyne, UK

**Keywords:** Social inclusion, Older people, Ageing, Systematic review, Health impact, Age-friendly environments

## Abstract

**Background:**

Many interventions have been developed to promote respect and social inclusion among older people, but the evidence on their impacts on health has not been synthesised. This systematic review aims to appraise the state of the evidence across the quantitative and qualitative literature.

**Methods:**

Eligible studies published between 1990 and 2015 were identified by scanning seven bibliographic databases using a pre-piloted strategy, searching grey literature and contacting experts. Studies were included if they assessed the impact (quantitatively) and/or perceived impact (qualitatively) of an intervention promoting respect and social inclusion on the physical or mental health of community-residing people aged 60 years and older. Titles and abstracts were screened for eligibility by one reviewer. A second reviewer independently screened a 10% random sample. Full texts were screened for eligibility by one reviewer, with verification by another reviewer. Risk of bias was assessed using standardised tools. Findings were summarised using narrative synthesis, harvest plots and logic models to depict the potential pathways to health outcomes.

**Results:**

Of the 27,354 records retrieved, 40 studies (23 quantitative, 6 qualitative, 11 mixed methods) were included. All studies were conducted in high and upper middle-income countries. Interventions involved mentoring, intergenerational and multi-activity programmes, dancing, music and singing, art and culture and information-communication technology. Most studies (*n* = 24) were at high or moderate risk of bias. Music and singing, intergenerational interventions, art and culture and multi-activity interventions were associated with an overall positive impact on health outcomes. This included depression (*n* = 3), wellbeing (*n* = 3), subjective health (*n* = 2), quality of life (*n* = 2), perceived stress and mental health (*n* = 2) and physical health (*n* = 2). Qualitative studies offered explanations for mediating factors (e.g. improved self-esteem) that may lead to improved health outcomes and contributed to the assessment of causation.

**Conclusions:**

Whilst this review suggests that some interventions may positively impact on the health outcomes of older people, and identified mediating factors to health outcomes, the evidence is based on studies with heterogeneous methodologies. Many of the interventions were delivered as projects to selected groups, raising important questions about the feasibility of wider implementation and the potential for population-wide benefits.

**Systematic review registration:**

PROSPERO registration number CRD42014010107

**Electronic supplementary material:**

The online version of this article (10.1186/s13643-018-0680-2) contains supplementary material, which is available to authorized users.

## Background

According to the *World Population Ageing* report [1], the world’s population aged 60 years and older is expected to increase to more than two billion by 2050; by 2030, one in six people will be 60 years or older A growing number of these older people live in urban environments, with particularly rapid increases in low- and middle-income countries [2]. The combination of population ageing and urbanisation places increasingly complexes demands on health and social care systems, raising significant challenges for welfare systems worldwide [3, 4].

The older population should be a net asset to society, but suitable policies and services in place will be required to realise this [4–6]. In the *Global Strategy and Action Plan on Ageing and Health*, published in 2016, the WHO advocated the development of physical and social settings that support older people to live independently and in good health for longer but also optimise health and wellbeing for the wider community [7]. *Age-friendly environments* aim to facilitate older adults’ access to opportunities for social interaction and engagement with cultural and social resources (e.g. libraries and green spaces) [8, 9].

A range of interventions have been developed to create age-friendly environments, based on eight different domains theorised by the WHO as having a potential impact on health and wellbeing [10]. One of these domains is respect and social inclusion, which has been considered of fundamental importance to older people in qualitative research [10–14] and in national and international policy [4, 6, 7, 15, 16]. Persistent disrespectful attitudes and misconceptions about older people and growing old are acknowledged as being a significant barrier to the development of good public health policies on ageing [7, 17]. They lead to negative perceptions of ageing (e.g. by disregarding the contribution older people make to society) and can negatively impact health and wellbeing in later life [18–20]. For instance, Levy et al. [21] have shown that older people who were exposed to negative age stereotypes were less likely to recuperate from disability than those exposed to more positive self-perceptions of ageing. Moreover, people who internalised negative age stereotypes sooner in life were more likely to experience cardiovascular events in the coming 38 years than those who had more positive self-perceptions of ageing [22].

The term social inclusion has explicit links with concepts such as equality, human rights and social cohesion, and it has focused on barriers that prevent people from participating meaningfully in society [19]. By focusing on goals rather than problems, the concept of inclusion adopts a positive approach [23, 24]. It is not merely the implied opposite of social exclusion but refers to the opportunities for individuals to cultivate social relationships, have access to resources and feel part of the community they live in [25, 26, 27]. Respect in relation to older people, meanwhile, refers to positive attitudes and behaviours towards the elderly, so that they may feel accepted, valued and appreciated by the community regardless of age [28].

Whilst many interventions to promote respect and social inclusion in older people have been developed, the evidence on their impacts on health and wellbeing has not been synthesised. One of the reasons for this limited synthesis owes to complexity of these interventions [29]. In this context, complexity may arise by the various interactions between the components that may be involved in the intervention and its context, and external factors. For instance, an intervention may indirectly improve the level of social engagement of older adults and, in turn, their wellbeing and quality of life [2]. The same intervention may also consist of relatively well-defined initiatives (e.g. reading activities) or may be a much more complex set of actions driven by policy (e.g. different reading activities in various schools). These are some of the reasons that make the assessment and synthesis of these interventions particularly challenging [30–39]. The WHO has identified synthesising the evidence on interventions promoting age-friendly environments as a key priority to establish what is known about the impacts of these [7]. Responding to this call, this systematic review attempts to synthesise the evidence of health impacts of interventions on respect and social inclusion in older people. It addresses the following research question: What is the empirical evidence on the impact on health and wellbeing of interventions which foster respect and social inclusion in community-residing older adults? The aims were to (i) identify and understand the health impacts of interventions that aim to promote respect and social inclusion among older people and (ii) to elucidate the complex pathways that may lead to improved health outcomes.

## Methods

We followed the Centre for Reviews and Dissemination’s guidance for undertaking reviews in healthcare [40]. The Preferred Reporting Items for Systematic Reviews and Meta-Analyses (PRISMA) guidelines informed our reporting [41]. The protocol was registered with the PROSPERO database [42], and a PRISMA checklist is available as Additional file 1.

The first step we took, before searching the literature, was to develop logic models depicting the possible multiple interacting pathways through which the interventions could affect health and wellbeing [35, 43–45], as recommended in the literature on evidence synthesis of complex interventions [36, 43, 46–50]. First, we conducted scoping work (which involved looking at existing literature reviews [51, 52] and key background literature [10, 14, 53, 54] on respect and social inclusion and age-friendly environments) to identify interventions, outcomes and mediating factors that were mentioned in relation to promoting respect and social inclusion in older people.

Two main types of interventions emerged: (1) intergenerational interventions and (2) information and communication technology interventions. For these two intervention types, we developed logic models at the start of the review process, based on the pathways mentioned in the literature; we then went on to adapt them over the course of the review process, to incorporate the additional information we identified. Please refer to Figs. 3 and 4, in the “Results”, for an example of the logic models for intergenerational interventions.

For interventions which were not identified from our scoping review, but which met our inclusion criteria (e.g. they were qualified as interventions promoting respect and social inclusion, such as music and singing activities), we generated logic models after studies were assessed for inclusion. These models were based on the information reported in the included studies about mediating factors and pathways. For further details on the synthesis process, please refer to the “Synthesis” section.

### Search strategy

We developed and piloted a search strategy designed to capture the most relevant evidence to address the research question. We searched eight electronic bibliographic databases: Scopus, MEDLINE, PsycINFO, CINAHL and Web of Science Core Collection: citation indexes (Social Sciences Citation Index, Science Citation Index, Book Citation Index–Science, Book Citation Index–Social Sciences and Humanities); the Web of Science Core Collection: citation indexes (Conference Proceedings Citation Index–Science, Conference Proceedings Citation Index–Social Science and Humanities); the Cochrane Library: Cochrane Reviews (Reviews and Protocols) and other reviews and trials (ProQuest Dissertations & Thesis). Searches comprised a combination of subject terms selected from the controlled vocabulary or thesaurus where possible (MEDLINE, CINAHL and PsycINFO) and a wide range of free-text terms. For the full electronic strategy used to search MEDLINE, see Additional file 2. Relevant systematic reviews were retrieved, and titles of individual studies were checked to see if they met the inclusion criteria.

We searched sources of grey literature including policy papers and reports from the following: the Joseph Rowntree Foundation (http://www.jrf.org.uk/), Age UK (http://www.ageuk.org.uk/), Age of Creativity (http://www.ageofcreativity.co.uk/), Alzheimer’s Association (http://www.alzheimers.org.uk/), InterGen (http://fromgeneration2generation.org.uk/), Beth Johnson Foundation (http://www.bjf.org.uk/) and Manchester Institute for Collaborative Research on Ageing (http://www.micra.manchester.ac.uk). We checked the list of references of potentially relevant papers included as full text if the title met the inclusion criteria. We also contacted topic experts to identify any additional data sources.

Searches were restricted to the English language as there were no resources for translation. We were interested in the literature relevant to contemporary social and political contexts of ageing and respect and social inclusion. The aim of our review was to identify evidence about interventions which could be implemented in the context of current efforts to promote age-friendly environments. We therefore chose the 1990 as the start date of our searches (up until January 2015, when the search was conducted) to coincide with the emergence of debates about, and initiatives aimed at, designing optimum community environments for ageing populations [55].

### Inclusion criteria

1. *Population*: Studies where at least 50% of participants were aged 60+ years were eligible for the review. Those where some of the population were younger than 60 years were included if the data for subgroups of older people (60+ years) could be disaggregated or where the average age was over 60.
2. *Interventions*: Any intervention aiming to improve respect and social inclusion in older people was included. Studies were included if they did not explicitly mention either term but where the purpose of the intervention was to improve community inclusion, social participation, sense of belonging, access to learning, cultural and social opportunities or social relationships in the community. We only included mentoring interventions where the aim was to engage older people in social activities with others within a group setting. By contrast, befriending interventions focus on improving the level of social support and decreasing loneliness through one-to-one interaction [56]. Because the latter is not a group- or community-based activity, it did not meet our inclusion criteria.
3. *Control groups*: Relevant comparison groups included (i) older people not exposed to the intervention being investigated, (ii) older people exposed to other forms of interventions included as *usual practice* and (iii) older people exposed to other interventions for respect and social inclusion. This criterion applied only to quantitative studies.
4. *Outcomes assessed quantitatively*: Health outcomes pertinent to the review included any measure of physical and mental health of participants, health-related quality of life and measures of wellbeing. Standardised outcome measures were defined as those supported by an academic reference and evidence of their psychometric properties. Non-standardised health outcome measures were defined as those developed by the authors for the purposes of the study. Although we recognised that cognitive function is a health outcome, through our logic models, outcomes related to cognitive function (e.g. memory and language attention) were included only if there was evidence that the intervention (e.g. Internet training) increased respect and social inclusion and that this led to the improved outcome. Likewise, outcomes related to autonomy and physical activity (e.g. posture, balance, muscle strength, stability and walking speed) were included only if there was evidence that the intervention (e.g. dancing classes) increased respect and social inclusion and that this led to the improved outcome.
5. *Setting*: Only studies conducted in community settings were included in the review. Studies that included groups from both community and institutionalised settings (e.g. nursing homes) were included if the community data could be disaggregated.
6. *Study design*: All empirical study designs including quantitative designs (randomised and non-randomised controlled studies, before and after studies), mixed methods design and qualitative designs were eligible for the review. Case studies (defined as “drawing on multiple sources of information to provide a broad evaluation of a specific project, program, or policy” ([52] p. 122) were only included if sampling techniques, data collection methods and results/analysis of health impacts could be ascertained.

### Screening and selection

Search results were downloaded into EPPI-Reviewer 4 software [57]. After removing duplicates, titles and abstracts were screened for eligibility by one reviewer (SR), using a pre-designed and piloted tool based on the inclusion criteria. A second reviewer (NKV) independently screened a 10% random sample of titles and abstracts. The level of agreement was checked using EPPI-Reviewer 4 software. This produced a reconciliation report showing that there was less than 2% disagreement out of 2736 papers independently coded by the two reviewers (SR and NKV). Disagreements were resolved through discussion or by recourse to a third reviewer (LO/DP/NB). One reviewer (SR) screened full-text papers for eligibility with 15% screened by another reviewer (LO/DP/NB) where there was uncertainty about the relevance for inclusion. Any discrepancies were resolved through discussion, and decisions for exclusion were documented.

### Data extraction and risk of bias assessment

A single reviewer (SR) conducted data extraction for included studies using separate pre-piloted forms for quantitative and qualitative evidence; one reviewer (DP/LO/NB) checked 15% of data extraction tables. Extracted information included (i) bibliographic details, (ii) study design, (iii) study participants including details of control groups for quantitative studies, (iv) aims and key features of the intervention, (v) outcomes and outcome measures, (vi) main results, (vii) main conclusions and (viii) key methodological issues. From the qualitative studies, we extracted participants’ own narratives and then summarised these *data* in a concise message in data extraction tables. The summary included information on factors (e.g. improved self-esteem) reported by older people on the impact of the intervention on their health and wellbeing.

All studies were critically appraised by one reviewer (SR). We assessed risk of bias (RoB) and methodological quality using different methods for quantitative and qualitative studies, as explained below. For shorthand, we reported the overall assessment of quality as RoB throughout this paper and we used it as preferred terminology [52]. In the summary tables (Additional files 4 and 5), we used a global assessment for quantitative and qualitative studies. This was used to facilitate reporting of the data in the summary tables and give an indication of the RoB among the different studies. As recommended by the literature [58], we incorporated the RoB assessments into the findings (please refer to the “Results” section). For the item-level RoB assessment for each study, please refer to Additional file 6 (quantitative studies) and Additional file 7 (qualitative studies). Case studies were assessed using an adapted version of Atkins & Sampson’s tool [59]. Quantitative studies and quantitative elements of mixed method studies were assessed using the Liverpool Quality Assessment Tools (LQATs) [60]. The forms include (i) selection procedures, (ii) baseline assessment, (iii) outcome assessment, (iv) analysis/confounding and (v) contribution of evidence towards the review question that are rated as strong, moderate or weak. Qualitative studies and qualitative elements of mixed methods studies were appraised using an adapted version of Harden et al. [61, 62] and Mays and Pope [63] tools. The form is divided into sections covering study context, methodology, use of strategies to increase reliability and validity and extent to which findings reflected participant perspectives and experiences. A global assessment of validity was made based on whether aspects of the study were clear, adequate or explicit using this scale.

### Synthesis

The broad focus of interventions fostering respect and social inclusion, and the heterogeneity across study designs and outcomes, precluded meta-analysis [42]. We therefore conducted a narrative synthesis [40, 64] comprising four elements:

1. We grouped and tabulated studies according to the type of intervention evaluated. A broad range of interventions were identified, including those based on (i) mentoring, (ii) intergenerational programmes, (iii) dancing, (iv) music and singing, (v) art and culture, (vi) information-communication technology and (vii) multi-activity programmes (e.g. health promotion). To facilitate reporting of the data in the summary tables (Additional files 4 and 5) and to give an indication of the potential RoB among the different studies, we ranked quantitative and qualitative studies based on a *global assessment* (from lower to higher RoB).
2. For each intervention category, we produced a narrative summary of findings, grouping studies according to whether they produced similar results, measured the same outcomes and/or shared a theoretical basis [64]. RoB was discussed in each narrative summary [58].
3. We used harvest plots to graphically represent the quantitative findings, including RoB for each intervention (Table 1). These plots represent an overall summary of the quantity, direction and strength of the evidence for the various health outcomes [47].
4. *Logic model development*:
  As explained earlier, based on scoping work, we generated logic models for (1) intergenerational interventions and 2) information and communication technology interventions. The initial construction of the logic models (pre-review) helped us to conceptualise possible outcomes and mechanisms through which interventions on social inclusion might produce effects on health outcomes. Successively, based on the evidence retrieved, we assessed whether the mediating factors and outcomes that we depicted in the initial logic models were supported by the evidence (see Figs. 3 and 4).
  *Diagram development*:
  Diagrams were developed during the narrative synthesis process. They represent a descriptive overview of the quantitative and qualitative evidence retrieved for each intervention type.
  The mediating factors included in the diagrams came from the participants’ own narratives that emerged from the qualitative studies (on how older people reported the impact of the intervention). They offer some explanations about possible mechanisms through which interventions on respect and social inclusion may impact on older people’s health (e.g. feeling valued). The diagrams also present the list of outcomes being studied by the qualitative and quantitative studies (including number of studies), and the effect for quantitative studies (see Figs. 5, 6, 7, 8, 9 and 10). We have not included the assessed RoB in these diagrams, as the RoB is presented in the harvest plot (Table 1).

**Table 1:**
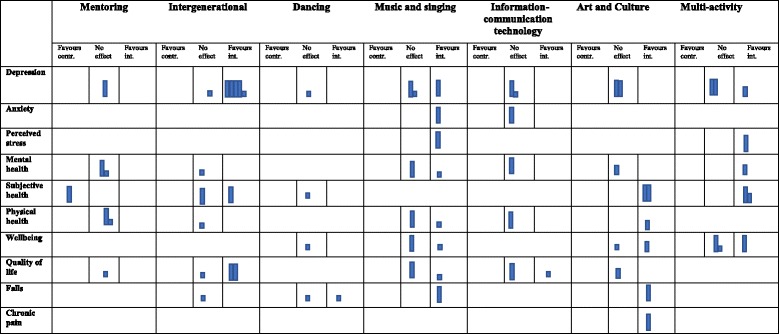
Harvest plot for interventions on respect and social inclusion in older people

Each solid bar represents a study. The height of the bar reflects the RoB assigned to that study (high bar*,* low/low-moderate RoB; medium bar*,* moderate RoB; low bar*,* moderate-high/high RoB), so that the strength of the evidence could be determined, and greater weight is given to conclusions from the most methodological robust and reliable studies. See “Methods” for assessing RoB in quantitative studies

## Results

### Study selection

**Fig. 1:**
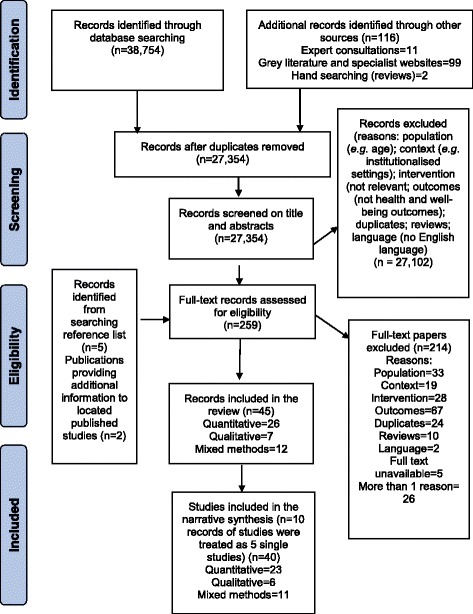
PRISMA flow diagram of the study selection process

Of the initial 27,354 references retrieved, 259 were filtered for full-text paper review after screening titles and abstracts. Of these, 45 records based on 40 studies (23 quantitative, 6 qualitative, 11 mixed methods) met the inclusion criteria of the review (Fig. 1). The PRISMA flow diagram of the study selection process is shown in Fig. 1.

### Study characteristics

**Fig. 2:**
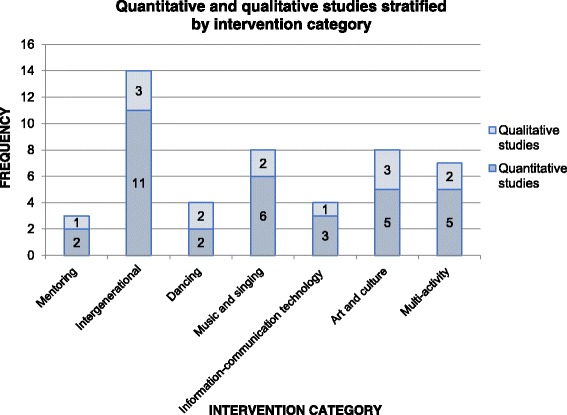
Quantitative and qualitative studies stratified by intervention category

Additional file 4 summarises the characteristics of the quantitative studies, and Additional file 5 presents the characteristics of the qualitative studies. Table 1 shows the harvest plot, which represents a brief overview of the strength of the quantitative evidence for the various health outcomes and the RoB of the studies. In Fig. 2, the number of qualitative and quantitative studies is stratified by intervention category (*n* = 40).

Studies using mixed methods designs contributed to both quantitative and qualitative evidence. Thirty-four studies provided quantitative evidence and 14 studies qualitative evidence. One study [65] contributed to both the mentoring and intergenerational interventions, and another [66] contributed to both singing and art and culture interventions.

#### Study design

Of the 34 studies reporting quantitative evidence, seven adopted individual or cluster randomised controlled trials (RCTs), with the rest using quasi-experimental designs—four were controlled before and after studies, seven were cluster or individual controlled studies and 15 were uncontrolled before and after studies. Studies reported a range of comparison/control groups including other interventions not related to respect and social inclusion (e.g. weekly recreational activities) (*n* = 2), usual care (e.g. through routinely available health, social and voluntary care services) (*n* = 2), other activities (e.g. hobbies) (*n* = 5) and older people selected from waiting lists (*n* = 2). One study used multiple comparison groups [67].

Most studies had only one follow-up assessment conducted between 2 weeks and 8 months after initiation of the intervention/baseline measurements.

Of the 14 studies reporting qualitative evidence, the methods used included the following: focus group discussions (*n* = 3), interviews (*n* = 7), a mix of focus groups and interviews (*n* = 2), diary writing (*n* = 1), observation (*n* = 1) and qualitative comments offered in response to open questions included in the questionnaires (*n* = 1).

#### Setting

All studies concerned higher and upper middle-income countries. Thirteen were from the UK; 13 from the USA; three from Japan; two each from the Netherlands, Australia, Canada and Brazil; and one each from Spain, Italy and China.

#### Population

The majority of studies included healthy older people aged between 60 and 95 years, with the exception of two studies that included older people with dementia [68, 69] and three studies that included older people with Parkinson’s disease [70–72].

Most studies comprised a majority of women, with only one study reporting an even balance between women and men [65] and one study including women only [73]. In most studies, participants were either volunteers currently involved with/interested in the programme or those recruited through fliers and letters. Study participants were also referred by general practices [74] or recruited from day centres [68] and community centres/groups [75, 76].

#### Delivery and frequency of contacts

Four studies included interventions delivered by peers [77–80], eight were led by the study participants themselves [65, 76, 81–86], one involved both professionals and students [87], four were led by study participants with some support from help desk and community centres [67, 88–90] and 19 studies were led by professionals [66, 69–75, 91–103].

The frequency of contact with participants varied, with most interventions being delivered on a weekly or other periodic basis (e.g. every 2 weeks). Most interventions lasted between 3 and 12 weeks, with a few lasting for extended periods (26 weeks [73], 30 weeks [66] and 3 years [96]). In one study, the intervention duration was not clear [85].

#### Outcomes

Impacts were reported on the following: depression (*n* = 20), subjective health (*n* = 7), mental health (*n* = 4), wellbeing (*n* = 8), physical health (*n* = 7), quality of life (*n* = 7), falls (*n* = 4), perceived stress and anxiety (*n* = 3) and chronic pain (*n* = 1). See Additional file 3 for an overview of the scales used for the quantitative studies in measuring outcomes. Most of the included studies used standardised scales, with only a few studies using non-standardised measures for subjective health [66, 79, 82, 83], falls [70, 76] and quality of life outcomes [78, 95].

#### RoB

Overall, 12 studies were rated as high and medium-high RoB [65, 69, 70, 72–74, 76, 78, 85, 95, 103, 104], 12 studies as moderate RoB [70, 71, 75, 80, 83, 84, 93, 94, 97, 98, 100, 102] and 21 as low or low-moderate RoB [66–68, 77, 79, 81, 82, 86, 87, 89, 90, 92, 96, 98–100, 105–107]. The main RoB issues with these studies included small sample size, poor selection of participants and differences observed between intervention and control group participants at baseline.

#### Mediating factors

Of the 14 studies reporting qualitative evidence, the most common mediating factors explored were the following: improved self-confidence and self-esteem, feeling valued, improved social relationships and interactions, reduction of social isolation, feeling of happiness and enjoyment and feeling more physically active.

### Development of logic models: pre and post review

**Fig. 3:**
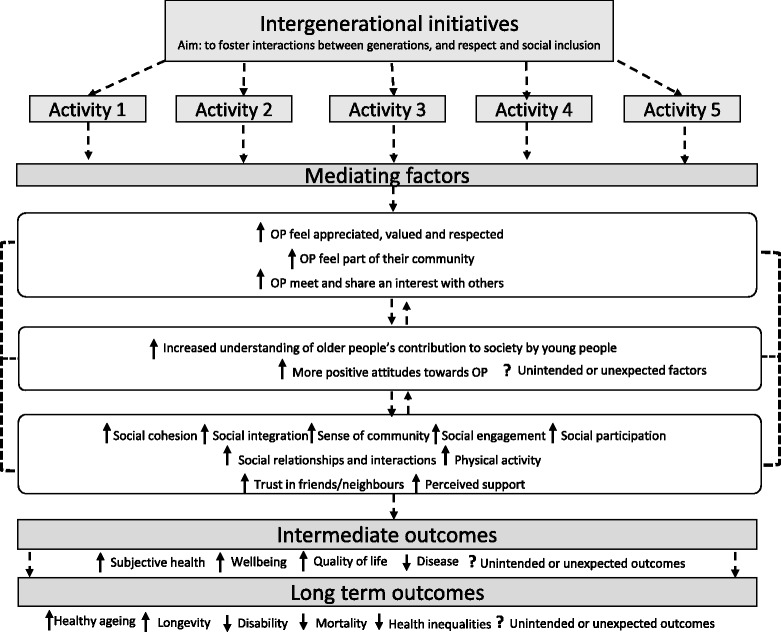
The logic model shows some possible outcomes and mediating factors based on scoping work. OP refers to older people; black dashed arrow represents a relationship/impact; ↓ decrease; ↑ increase; (?) symbol means neutral/do not know

To illustrate how the logic models evolved thorough the review process, Fig. 3 shows the logic model that we initially developed for intergenerational interventions (pre review).

**Fig. 4:**
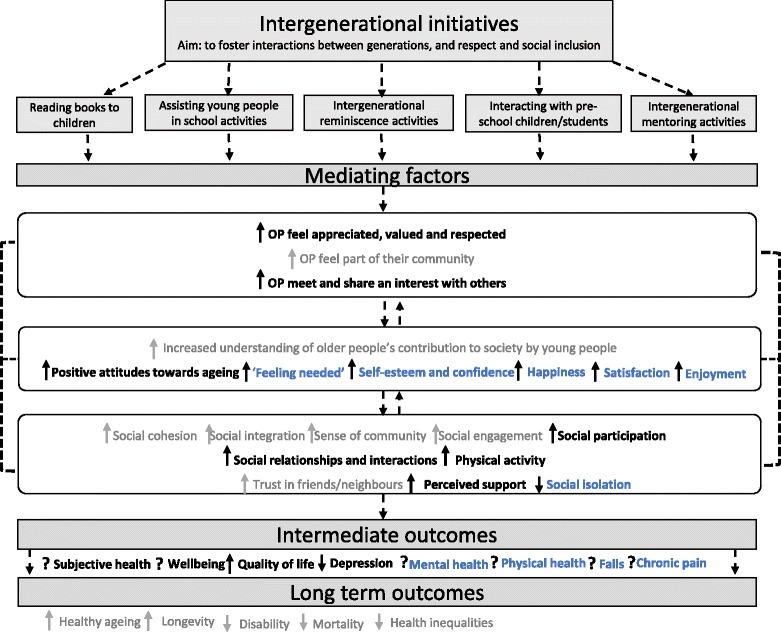
In bold are the mediating factors and outcomes that have been studied by the quantitative and/or qualitative studies. In blue are the additional mediating factors and outcomes identified in this review. OP refers to older people; black dashed lines represent a relationship; ↓ decrease; ↑ increase; (?) symbol means neutral/do not know/evidence is inconsistent

Based on the evidence retrieved, we assessed whether the hypothesised mediating factors and outcomes were supported by the evidence. As shown in the final version of the logic model (Fig. 4), through the review, we were able to identify some of the activities that constitute intergenerational interventions (e.g. reading books to children and assisting young people in school activities). From the quantitative evidence, which looked at the impact of the interventions, we generated some additional outcomes (e.g. depression). From the qualitative data, which provided information on how interventions might work according to older people’s narratives, we generated some additional mediating factors (e.g. feeling valued) that could be involved in the process of improving health outcomes.

### Results by intervention category

For each intervention category, the number of studies, the type of study design and the RoB for each study are presented with a summary of the main findings (both quantitative and qualitative evidence). For a quick overview of the strength of the quantitative evidence and the RoB of the studies, please refer to the harvest plot (Table 1). For a more detailed explanation of the findings and RoB for each study, please refer to Additional file 4 (quantitative studies) and Additional file 5 (qualitative studies). The item-level RoB assessment for each study can be found in Additional file 6 (quantitative studies) and Additional file 7 (qualitative studies).

Whilst many studies reported stratification by socio-economic status, education and gender at baseline, few reported sub-analyses of health outcomes by age, ethnic or education of older people.

#### Mentoring interventions

Two quantitative studies looked at mentoring (Additional file 4): an individual RCT of a community-based mentoring service programme rated as low-moderate RoB [77] and an uncontrolled before and after study of an intergenerational mentoring programme rated as high RoB [65]. Differences observed between comparison groups at baseline [77] and small sample sizes [65] made it difficult to interpret the results.

One study found no effect on depressive symptoms (mean difference (MD) = 0.2, *p* = 0.29) [77], and although it showed a significant improvement in subjective health at 6-month follow-up (MD = − 0.09, *p* < 0.01), this improvement was significantly less than controls (MD = − 0.1, *p* < 0.01) [77]. Two studies found no effect on mental health (MD = 0.8, *p* = 0.48 [77]; MD and *p* values not reported in the study by Ellis [65]) and physical health (MD = 0.1, *p* = 0.90 [77]; MD and *p* values not reported in the study by Ellis [65]). A further study did not observe an effect of mentoring on quality of life (MD and *p* values not reported in the study by Ellis [65]).

**Fig. 5:**
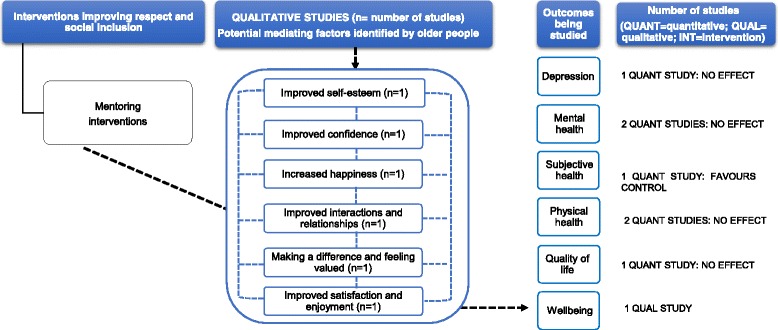
This diagram shows an overview of the outcomes (depression, mental health, subjective health, physical health, quality of life and wellbeing) that have been studied by the qualitative and quantitative studies (including number of studies), the effect for quantitative studies and the possible mechanisms for these effects as suggested by the qualitative evidence. The dashed arrows that go from the mediating factors to the outcomes indicate solely that according to some participants’ narratives, these factors may contribute to an improvement in health outcomes. See Additional files 4 and 5 for a summary of the studies, and the harvest plot (Table 1), which graphically represents the overall summary of the quantity, direction and strength of the quantitative evidence for the various health outcomes

One qualitative study included a mentoring programme, where older people acted as mentors for pre-school children [85] (Additional file 5). It included limited reporting of analysis, sampling and results. From the older people’s narratives, mentoring children was reported to help participants going through difficult times in their lives and to enhance their physical and mental wellbeing. Reported factors that might lead to an improvement in wellbeing were the following: improved self-esteem, satisfaction, confidence, interactions and relationships and feeling valued (Fig. 5).

#### Intergenerational interventions

Intergenerational studies included (i) mentoring initiatives [68, 81], (ii) interventions based on service-learning pedagogy [87], (iii) school initiatives [76, 82, 104], (iv) reading initiatives [83, 84], (v) reminiscence initiatives [75] and (vi) interventions involving reading and drawings [103].

Eleven quantitative studies were included Additional file 4): one individual RCT [76], one cluster RCT [81], two cluster controlled trials [83, 84], two controlled before and after studies [82, 87] and five uncontrolled before and after studies [65, 68, 75, 103, 104]. Seven were judged as low-moderate RoB, with four being moderate-high RoB [65, 76, 103, 104]. The main weaknesses of these studies were small sample size (*n* = 2) [103, 104] and lack of a control group (*n* = 2) [103, 104]. Reporting of analysis was limited in three studies [65, 76, 104].

Five studies found a significant effect on depression scores (reduction of 62% within 2 weeks after the completion of the programme: MD = 1.86, *p* value not reported in the study [68]; reduction of 26.3% obtained in the post-treatment evaluation: MD = 3.53, *p* < 0.001 [87]; reduction of 18.5% at 2-year follow-up: MD = 0.94, *p* < 0.001 [82]; reduction of 14%: MD = 0.31, *p* < 0.10 [84]; reduction of 16.64% at 68-week follow-up: MD and *p* value not reported in the study [104]), whilst one study found no effect at 8-week follow-up (MD = − 0.97, *p* = 0.3) [103].

One study showed a significant favourable effect on self-rated health scores at 21-month follow-up (*p* < 0.01; MD not reported in the study by Fujiwara et al. [83]), whilst one study did not find an effect at 4-month follow-up (*p* = 0.554) [81]. For quality of life scores, two studies showed some indication of an effect (an increase of 4.4% in the subscale of past, present and future activities after the completion of the programme: MD = − 0.65, *p* = 0.05 [75]; an increase of 7%: MD = − 1.91, *p* value not reported in the study by Chung [68]). One study (high RoB) did not observe an effect on quality of life, physical health and mental health [65]. In one study, participants experienced a non-significant decrease of more than 50% in falls rates at 4–8-month follow-up (*p* = 0.17) [76].

**Fig. 6:**
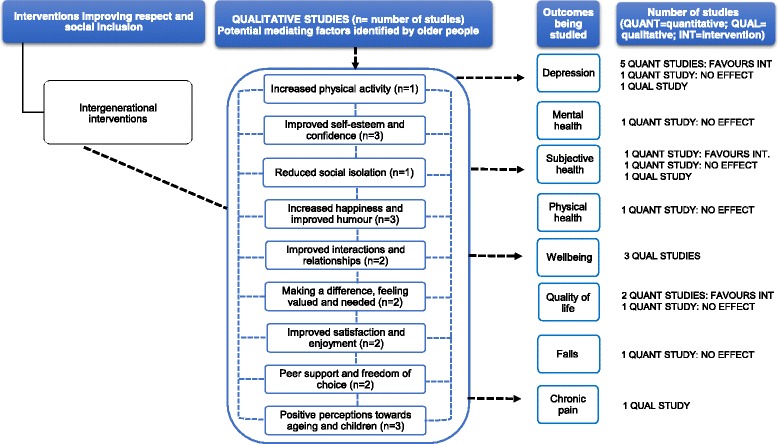
This diagram shows an overview of the outcomes (depression, mental health, subjective health, physical health, wellbeing, quality of life, falls and chronic pain) that have been studied by the qualitative and quantitative studies (including number of studies), the effect for quantitative studies and the possible mechanisms for these effects as suggested by the qualitative evidence. The dashed arrows that go from the mediating factors to the outcomes indicate solely that according to some participants’ narratives, these factors may contribute to an improvement in health outcomes. See Additional files 4 and 5 for a summary of the studies, and the harvest plot (Table 1), which graphically represents the overall summary of the quantity, direction and strength of the quantitative evidence for the various health outcomes

Three qualitative studies, of low-moderate RoB, were included (Additional file 5). Participants’ narratives identified some factors mediating the impact of wellbeing, subjective health and depressive mood [85, 86, 89] (Fig. 6). These included the following: improved self-esteem and confidence, enjoyment and satisfaction and happiness; improved interactions and relationships with others; feeling valued; and positive perceptions towards ageing and children. Older people’s narratives reported a perceived enhanced emotional and physical wellbeing and subjective health [85, 86, 89]. In a study conducted by De Souza [86], the female participants reported that the project helped to alleviate their depressive moods and to improve their overall wellbeing and humour.

#### Dancing interventions

Two quantitative studies were included (Additional file 4): an individual RCT [71] and an individual controlled trial [70]. They were both rated as high and moderate RoB [70, 71] due to differences between control and intervention groups in the depression levels at the outset of the study [71] and small samples [70, 71].

One study showed significant reduction in depression scores [71] (older people with Parkinson’s disease: MD = 0.26, *p* = 0.001; older people without Parkinson’s disease: MD = 0.52, *p* = 0.001). Neither study found an effect on wellbeing and subjective health between 2-week and 68-month follow-up [70]. Findings were mixed for falls rates, with one study showing a significant reduction in falls (MD and *p* values not reported in the study by Hackney et al. [71]) and the other showing no effect [70].

**Fig. 7:**
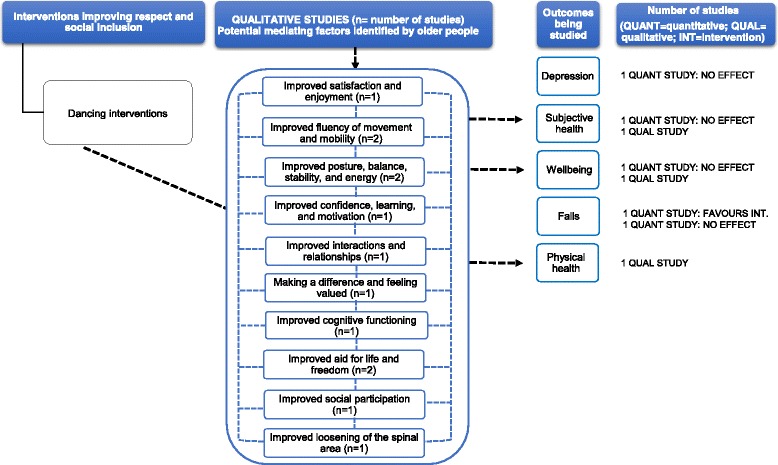
This diagram shows an overview of the outcomes (depression, subjective health, wellbeing, falls and physical health) that have been studied by the qualitative and quantitative studies (including number of studies), the effect for quantitative studies and the possible mechanisms for these effects as suggested by the qualitative evidence. The dashed arrows that go from the mediating factors to the outcomes indicate solely that according to some participants’ narratives, these factors may contribute to an improvement in health outcomes. See Additional files 4 and 5 for a summary of the studies, and the harvest plot (Table 1), which graphically represents the overall summary of the quantity, direction and strength of the quantitative evidence for the various health outcomes

Two qualitative studies provided context to the relationship between dancing and subjective and physical health, subjective health and wellbeing [70, 72] (Additional file 5). The main weaknesses of the studies included limited reporting of sampling, analysis and results. Participants’ narratives identified some factors mediating the impact of physical health, subjective health and wellbeing, and these (Fig. 7) comprised the following: improved satisfaction, enjoyment and confidence; improved fluency, dynamics of movement and mobility; improved social interactions; and feeling valued. Older people talked about how the programme made them *feel better*, giving them a sense of wellbeing [72], and made them feel good and capable despite some health difficulties [70].

#### Music and singing interventions

There were six quantitative and two qualitative studies that explored the impacts of music [93, 101] and singing initiatives [66, 69, 91, 94, 102, 108]. The six quantitative studies included (Additional file 4) the following: a cluster RCT [107], an individual RCT [66], a controlled before and after study [93] and three before and after uncontrolled studies [69, 92, 94]. Three were judged as low-moderate RoB [66, 92, 107], two as moderate RoB [93, 94] and one as high RoB [69]. The main issues were short follow-up [69, 94], small sample size [69, 94] and poor adjustment for potential confounders [93].

With regard to psychological outcomes, one study [91] found a significant reduction of 36.6% in depression scores at 3-month follow-up (MD = − 1.52, *p* < 0.01) and of 12.5% at 6-month follow-up (MD = − 0.53, *p* = 014). The same study [91] found a significant reduction of 31.1% in anxiety scores at 3-month follow-up (MD = − 1.78, *p* < 0.01). Two studies showed no effect on reduction in depressive symptoms at 12-month [66] and 8-week [94] follow-up. One study showed a reduction of 27.3% in perceived stress scores (MD = 2.58, *p* < 0.001) [92].

Two studies found a positive effect on mental health. One study showed a significant improvement of 9.4% in mental health-related quality of life scores (MD = 4.77, *p* < 0.01) at 3-month follow-up and of 5% at 6-month follow-up (MD = 2.35 *p* = 0.05). Another study found an improvement of 14.3% in mental health scores (vitality subscale: MD = 10.4, *p* = 0.03) at 8-week follow-up [94].

Two studies found a positive effect on physical health. One study showed an improvement of 14.3% in the vitality subscale (vitality subscale: MD = 10.4, *p* = 0.03) at 8-week follow-up [94]. Another study found an increase of 9.03% in physical health scores (MD = 0.72, *p* < 0.01) [66] at 12-month follow-up.

For quality of life and wellbeing, results were mixed: one study [93] found an improvement in two components of the wellbeing and quality of life scale (an increase of 14% in control: MD = 1.15, *p* = 0.0001; an increase of 7.6% in pleasure: MD = 0.8, *p* = 0.0001) at 9-month follow-up, and the other study found no effect [69]. One study showed a significant reduction of 104% in falls rates (MD = − 0.32, *p* < 0.05) [66] at 12-month follow-up.

**Fig. 8:**
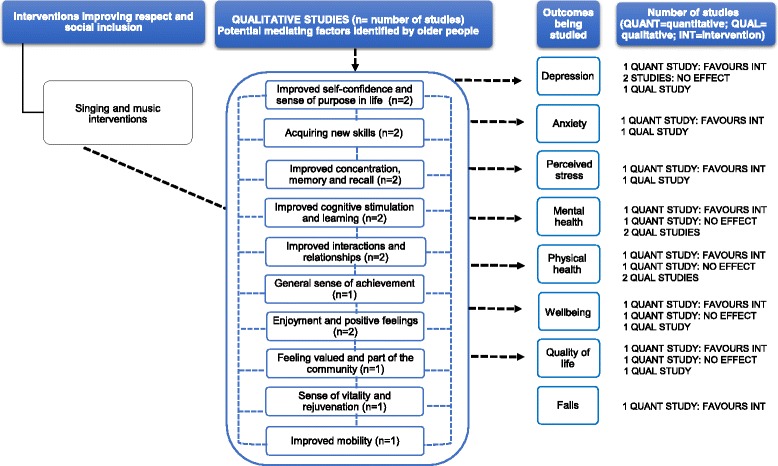
This diagram shows an overview of the outcomes (depression, anxiety, perceived stress, mental health, physical health, wellbeing, quality of life and falls) that have been studied by the qualitative and quantitative studies (including number of studies), the effect for quantitative studies and the possible mechanisms for these effects as suggested by the qualitative evidence. The dashed arrows that go from the mediating factors to the outcomes indicate solely that according to some participants’ narratives, these factors may contribute to an improvement in health outcomes. See Additional files 4 and 5 for a summary of the studies, and the harvest plot (Table 1), which graphically represents the overall summary of the quantity, direction and strength of the quantitative evidence for the various health outcomes

Two qualitative studies at low-moderate RoB gave context to the relationship between singing and music initiatives and the health outcomes [102, 105] (Additional file 5). Older people reported that music-making activities resulted in a better quality of life (e.g. enjoyment), mental health benefits (e.g. ability to cope effectively with stress) and physical health (e.g. good for asthma and breathing) [102, 105]. Participants’ narratives identified some factors mediating the impact of various health outcomes (depression, anxiety, perceived stress, mental health, physical health, wellbeing and quality of life). These included improved confidence, concentration and sense of achievement; feeling valued; and improved interactions with others (Fig. 8).

#### Information-communication technology interventions

Three quantitative studies were included (Additional file 4): two individual RCTs [67, 95] (low and moderate RoB) and one controlled before and after study [78] (moderate-high RoB).

Three studies found a non-significant reduction in depression scores (MD = − 1.4, *p* = 0.56 [67]; − 0.12 decrease on a 0–15 scale, *p* value not reported in the study by Woodward et al. [95]; 0.2 increase on a 0–15 scale, *p* value not provided in the study by Woodward et al. [78]). One study [67] found non-significant reduction in anxiety scores (MD = − 0.25, *p* = 0.56), improvement in mental health (MD = 1.03, *p* = 0.10) and physical health (MD = 2.63, *p* = 0.14). Findings were mixed for the two studies looking at quality of life outcome scores, with one intervention showing an improvement (4.99 increase on a 16–112 scale, *p* < 0.05) [95] at 6-month follow-up and the other showing no effect (6.1 increase on a 16–112 scale, *p* value not provided in the study by Woodward et al. [78]).

**Fig. 9:**
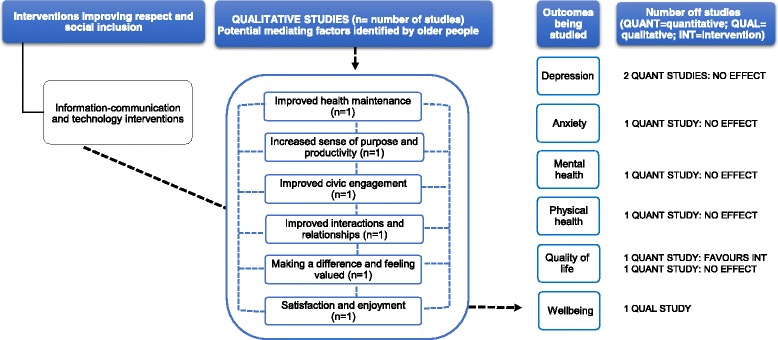
This diagram shows an overview of the outcomes (depression, anxiety, mental health, quality of life and wellbeing) that have been studied by the qualitative and quantitative studies (including number of studies), the effect for quantitative studies and the possible mechanisms for these effects as suggested by the qualitative evidence. The dashed arrows that go from the mediating factors to the outcomes indicate solely that according to some participants’ narratives, these factors may contribute to an improvement in health outcomes. See Additional files 4 and 5 for a summary of the studies, and the harvest plot (Table 1), which graphically represents the overall summary of the quantity, direction and strength of the quantitative evidence for the various health outcomes

One qualitative study (moderate RoB) [80] reported a perceived improvement in wellbeing (Additional file 5). Study participants related their enhanced sense of wellbeing acquired from using information-communication technology (ICT) to an increased sense of purpose and enjoyment to their lives. Some older people reported the programme served as a medium for strengthening existing relationships. Others mentioned that having ICT as a common interest brought them closer to family members. Other factors mediating the impact of wellbeing included improved health maintenance, satisfaction, civic engagement and feeling valued (Fig. 9).

#### Art and culture interventions

Five quantitative studies were included (Additional file 4): one individual controlled trial [66] and four before and after uncontrolled studies [74, 96, 98, 109]. Studies were rated as low-moderate RoB (*n* = 2) [66, 96], moderate RoB (*n* = 2) [97, 98] and high-moderate RoB (*n* = 1) [74]. Study weakness included small sample size, no control group and adjustment for known confounders not reported.

With regard to psychological outcomes, two studies showed non-significant reductions in depression scores at 2-year follow-up (MD = 0.7) [96] and at 12-week follow-up (MD = − 0.7) [66]. One study showed no effect on mental health at 1-month follow-up (MD = − 2.8, *p* = 0.154) [98].

One study found a significant improvement of 21.1% in physical health scores (MD = − 11.9, *p* = 0.030) [98] at 1-month follow-up. Two studies found a significant effect on subjective health scores (an increase of 14% (MD = − 0.4, *p* < 0.10) [96] at 2-year follow-up; an increase of 9% (MD = 0.72, *p* < 0.01) [66] at 12-week follow-up).

In terms of wellbeing scores, one study found a significant effect (an increase of 27.6%: MD = − 20.2, *p* = 0.002) [98], and one found no effect (MD = − 6) [74]. One study did not find an effect on health-related quality of life scores (MD = not reported, *p* = 0.88) [97]. One study showed a significant reduction in falls rate (reduction of 104%, *p* < 0.05) [66] and another on chronic pain scores (reduction of 23%: MD = 0.5, *p* < 0.05) [96].

**Fig. 10:**
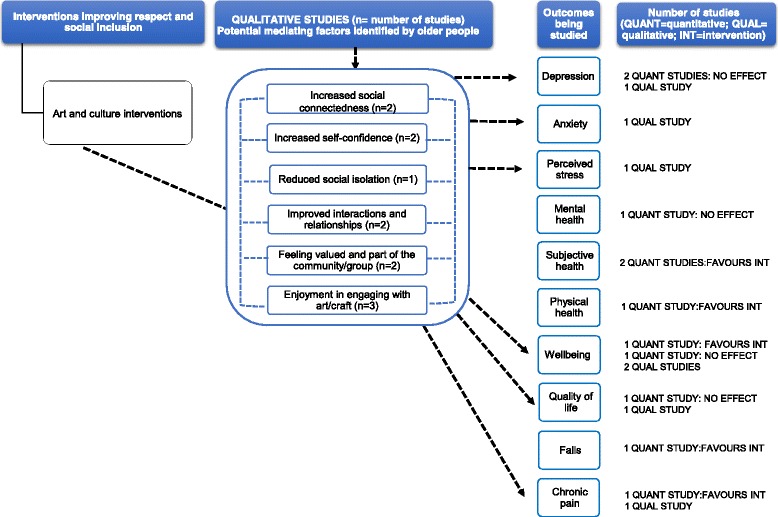
This diagram shows an overview of the outcomes (depression, anxiety, perceived stress, mental health, subjective health, physical health, wellbeing, quality of life, falls and chronic pain) that have been studied by the qualitative and quantitative studies (including number of studies), the effect for quantitative studies and the possible mechanisms for these effects as suggested by the qualitative evidence. The dashed arrows that go from the mediating factors to the outcomes indicate solely that according to some participants’ narratives, these factors may contribute to an improvement in health outcomes. See Additional files 4 and 5 for a summary of the studies, and the harvest plot (Table 1), which graphically represents the overall summary of the quantity, direction and strength of the quantitative evidence for the various health outcomes

Three qualitative studies were included (Additional file 5). Participants’ narratives provided context to the association of art and culture interventions with health outcomes (depression, anxiety, perceived stress, wellbeing, quality of life and chronic pain). Older people described how creative work helped them to reduce their feelings of stress and anxiety and to overcome some health limitations (e.g. depression) [96, 110]. They also reported feeling more socially and physically active and feeling more relaxed [96]. Other factors mediating the impact included reduced social isolation, increased self-confidence, social connectedness, improved social interactions and feeling valued (Fig. 10).

#### Multi-activity interventions

Five quantitative studies were included: an individual RCT [99], two individual controlled trials [73, 79] and two before and after uncontrolled studies [100, 106] (Additional file 4). Studies were rated as low to moderate RoB (*n* = 3) [79, 99, 106], moderate RoB (*n* = 1) [100] and moderate-high RoB (*n* = 1) [73]—due to no random allocation of the intervention or control groups and convenience sampling methods.

Multi-activity interventions included (i) projects to encourage older people to participate in various activities organised in the city [99], (ii) creative exercise and/or cultural activities wherein older people were guided by peers [100], (iii) regular gatherings in neighbours’ homes and interactions with others [106], (iv) social clubs and exercise programmes [79, 90] and (v) regular meetings to discuss health information topics including people’s feelings and health [73].

Findings for psychological outcomes were mixed. One study found a significant reduction of 13.4% in depression scores at 6-month follow up (MD = 0.60, *p* < 0.02) [100] and of 11.6% at 12-month follow-up (MD = 0.56, *p* < 0.05) [100]. By contrasts, two studies did not find an effect (MD = 0.03 at 9-month follow-up [106]; MD = 0.4 at 6-month follow-up [99]). One study showed a significant improvement of 6.24% in mental health scores (MD = 3, *p* < 0.005) [100] but at the first follow-up only (6 months). One study found a significant reduction of 11.7% in perceived stress scores (MD = 2.23, *p* < 0.001, at 9-month follow-up) [106].

Two studies found a positive effect on subjective health scores (an increase of 5.15% (MD = 0.37, *p* < 0.01) at 3-month follow-up [79]; an increase of 4.2% (MD = 1.57, *p* = 0.06) at 12-month follow-up [100]). One study found a positive effect on wellbeing scores (an increase of 9%: MD = − 1.9, *p* = 0.039 [99]) at 6-month follow-up. By contrast, two studies did not find an effect on wellbeing (MD = 0.42 [79]; MD = 1.47 [73]).

**Fig. 11:**
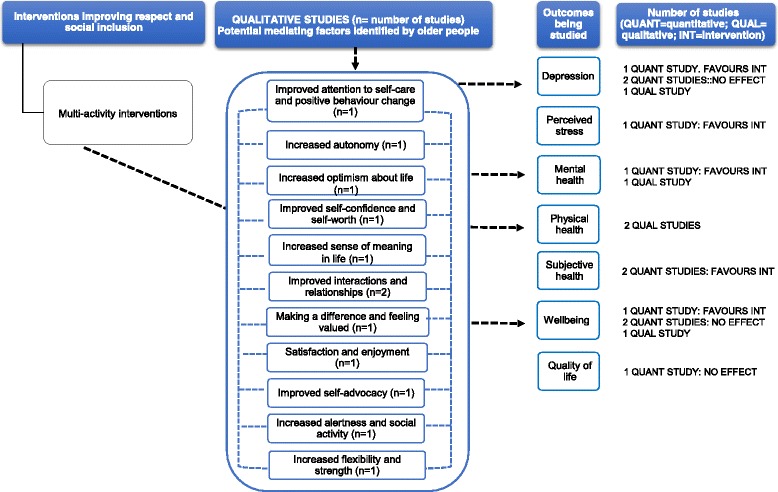
This diagram shows an overview of the outcomes (depression, perceived stress, mental health, physical health, subjective health, wellbeing and quality of life) that have been studied by the qualitative and quantitative studies (including number of studies), the effect for quantitative studies and the possible mechanisms for these effects as suggested by the qualitative evidence. The dashed arrows that go from the mediating factors to the outcomes indicate solely that according to some participants’ narratives, these factors may contribute to an improvement in health outcomes. See Additional files 4 and 5 for a summary of the studies, and the harvest plot (Table 1), which graphically represents the overall summary of the quantity, direction and strength of the quantitative evidence for the various health outcomes

Two qualitative studies were included (Additional file 5). Participants’ narratives gave insight on the relationship between multi-activity interventions and reduction in depression [100], wellbeing [90] and improved physical health [90, 100] (Fig. 11). These included (i) improved attention to self-care, self-worth and enjoyment; (ii) improved social interactions; and (iii) and feeling valued. Older people reported perceived psychological and physical health benefits including *feeling better*, increased flexibility and strength.

## Discussion

This is the first systematic review to report on the health impacts of interventions promoting respect and social inclusion in community-residing older people. By drawing on data from both quantitative and qualitative studies, it uniquely furthers our understanding of the pathways that may lead to improved health and wellbeing.

### Summary of findings

Intergenerational and music and singing interventions (for which there was the largest evidence base: 14 studies for intergenerational initiatives and eight studies for singing and music interventions), art and culture and multi-activity interventions showed an overall positive effect on various health outcomes. Quantitative studies identified impacts on depression (*n* = 3), wellbeing (*n* = 3), subjective health (*n* = 2), quality of life (*n* = 2), perceived stress and mental health (*n* = 2) and physical health (*n* = 2). In contrast, due to a paucity of evidence for mentoring, dancing and ICT interventions, it was not possible to make a judgement of the impact on health outcomes.

Qualitative studies identified some mediating factors (e.g. improved self-esteem) that may lead to improvements in health outcomes. For instance, in most intergenerational initiatives (Fig. 6), older people were regularly involved in assisting young people in school activities (e.g. math problems), and reading books to pre-school children. It appears that regular interaction with young people may have led older people to feel more valued, included, and appreciated. As a result, older people reported enhanced subjective health.

### Findings in relation to the literature

A number of reviews have explored the links between different social aspects of ageing and health outcomes [111–119]. For instance, the Centre for Reviews and Dissemination (CRD) [120] has provided a summary of several systematic reviews of interventions addressing social isolation and loneliness in older people [111–116]. Although related, social isolation and loneliness differ from the concept of social inclusion adopted in this study. Social isolation mainly refers to the quantity and quality of social support or contact received by others [111]. The same applies for loneliness, which is defined as “a subjective concept resulting from a perceived absence or loss of companionship” [111]. Dickens et al. [111] looked at both one-to-one and group-based interventions targeting social isolation and loneliness. They found that group-based interventions (e.g. psychosocial activity group) were more likely to have a positive effect on at least one of the four social health subdomains if compared with the one-to-one interventions (e.g. volunteer home visiting intervention). As we were interested in interventions focusing on making people valued and part of the community, we included only group-based interventions.

Previous reviews have looked at health impacts of specific interventions, including music [121], computer and internet training [116], dancing [122, 123] and gender-based interventions [117]. In her scoping review, Milligan et al. [117] assessed the evidence for the impacts of gendered social interventions (*Men’s Sheds*) on the health and wellbeing of older men. There are some similarities with our review, in terms of the complexity and typology of interventions as well as the approach used to synthesise the evidence of these studies. Firstly, although very specific, Men’s Sheds interventions aim to encourage older men to meet and socialise, learn new skills and take place in a community setting. Secondly, Milligan et al. [117] included qualitative and quantitative evidence and found that interventions were heterogeneous particularly in terms of (i) methodology, (ii) outcome measures and (iii) variety of activities within the interventions. Thirdly, the main weakness of their studies included low sample representativeness and lack of control group. All these aspects contributed to challenges in synthesising evidence of the health benefits of these interventions, as in our review.

Other reviews have focused on a diverse range of interventions but examined the effect on specific health outcomes. For instance, Lafortune et al. [118] examined the effectiveness and cost-effectiveness of various interventions promoting healthy behaviours (e.g. diet, physical activity/inactivity, alcohol, smoking, cognitive activity and risk reduction relating to loneliness and isolation) and their impact on primary prevention or delay of cognitive decline or dementia. They reported that interventions promoting social participation were associated with an overall positive impact on cognitive outcomes. Similar to our review, they found that reading to children in schools or art sessions may improve social, mental or physical health of older people. Disadvantaged groups were also underrepresented, with many studies being heterogeneous in intervention types and/or outcome measures.

The scope of our review includes all types of interventions that aimed to improve respect and social inclusion in older people and assessed associated impacts on health and wellbeing. Only six studies [66, 67, 71, 76, 83, 95], included in the reviews mentioned above [111, 115, 116, 118, 119, 121, 123], were directly concerned with older people and with the definition of interventions promoting respect and social inclusion adopted in this study.

### Strengths and limitations

We adopted a comprehensive and systematic approach for reviewing the evidence on a complex topic. All study designs were considered, and our inclusive approach allowed us to include a range of intervention types and health outcomes and positive and negative effects, which we attempted to summarise in the harvest plot (Table 1). The search was restricted to studies published in English, and this may have introduced language bias since significant results are more likely to be published in English-language journals than those reporting non-significant results [124]. This may also explain why all included studies concerned higher and upper middle-income countries. Due to the heterogeneity of the included studies, we used a narrative synthesis approach to summarise the findings of studies of this review. We were therefore unable to quantitatively assess publication bias by, for example, looking for funnel plot asymmetry [40].

We used the LQATs to assess the RoB of quantitative studies. LQATs have been used in a number of previous systematic reviews [125, 126] and have been critically examined in relation to other quality appraisal tools [127]. Qualitative studies were appraised using established criteria related to reliability and validity of findings developed by Harden et al. [62] and Mays and Pope [63]. Whilst these tools have been used extensively, the global assessment approach that we used was not previously validated in the appraisal of the original tools. An important limitation of this systematic review is that the majority of the review work was conducted by one reviewer, and some eligible studies may have been missed [40].

By drawing on both quantitative and qualitative evidence, we have explored both the effectiveness of relevant interventions (primarily quantitative evidence) and the mediating factors to improve health and wellbeing outcomes (primarily qualitative evidence). We feel that this approach has led to a better overall understanding of the current evidence base on interventions on respect and social inclusion in older people than would not have been possible using either quantitative or qualitative evidence alone [35, 128]. Qualitative studies helped us to understand some of the complexity of the wide range of components of each intervention and to clarify some aspects of the complexity related to *how* and *why* interventions may work or not work [29, 46, 128]. By doing so, qualitative studies contributed to the assessment of causality.

### Public health and policy implications

Many of the interventions reviewed were delivered as projects to selected groups, raising important questions about feasibility of wider implementation and potential for population benefits [129, 130]. Our findings suggest that studies mainly relied on people who volunteered. Since these people are generally more willing to participate in the community, they may not be representative of the entire population, particularly of *hard-to-reach* older people (e.g. those experiencing social exclusion, isolation, poverty and health problems). Services and other initiatives promoting respect and social inclusion (and similar approaches) should be provided to every older person who stands to benefits from these, and good policies in place should remove the barriers that limit people in most need (e.g. marginalised groups) in accessing these interventions [4].

### Research implications

Many of the interventions included in this review were implemented through weekly and monthly activities (e.g. reading books to children). These activities were facilitated by professionals, students, peers or older people themselves and took place in community centres and schools. Further research should assess the cost-effectiveness of these interventions (including when applied at greater scale in response to population need), particularly those that have shown a positive health impact (singing and music, intergenerational interventions, art and culture and multi-activity interventions).

Whilst age, gender, education, ethnic and socio-economic status of older people were recorded in the data extraction tables, only two studies reported them, and overall, the quantity and heterogeneity of the evidence precluded useful analysis of differential effects. Newman et al. [104] explored the effect of an intergenerational programme in reducing perceived depression by education level and age. The study showed that older people in the lower education group (high school) experienced an increase of 1.61% in perceived depression at 6–8-week follow-up. By contrast, those in the higher education group (college) reported a decrease of 26.42% in perceived depression at 6–8-week follow-up. About the effects by age, the older group (70 and over) experienced a decrease of 24.27% in perceived depression at 6–8 weeks post test, whilst the younger group (60 and over) reported an increase of 4.77% in perceived depression at 6–8 weeks post test (Additional file 4). One qualitative study [86] has reported differences in perceived impacts between males’ and females’ narratives, such that whilst male and female participants reported an improvement in subjective health, only females reported that the project helped them to alleviate their depressive moods and to improve their overall wellbeing and humour. Looking at differential effects would be a potentially important topic for future analyses as the evidence base expands.

Fifteen studies lacked a control group, making it difficult to be confident that self-reported improvements in psychological outcomes, subjective health, wellbeing and quality of life were directly attributable to the actual interventions. When interpreting our findings, we should note that some studies may have shown a favourable effect as a result of the Hawthorne effect, whereby participants’ awareness of being observed may have engendered beliefs about researcher expectations [131].

Considering these challenges, more robust evidence is needed to provide more certain/significant answers about the impact of these interventions. Future studies should (i) take advantage of natural policy experiments fostering respect and social inclusion, (ii) design better in-depth qualitative studies to explore the influence of context and mediating factors, (iii) use rigorous methodologies including randomised designs and (iv) assess whether the most promising interventions are also the most cost-effective.

## Conclusions

In the context of an increasing ageing population, it is important to establish what is known about the impacts of interventions that have the potential to improve older people’s health. This review suggests that music and singing, intergenerational initiatives, art and culture and multi-activity interventions may positively impact on wellbeing, subjective health, quality of life and physical and mental health. From the qualitative studies, there was evidence of plausible mediating factors including strengthened social relationships, improved self-confidence and self-esteem, feeling valued, reduction of social isolation and feeling more physically active. However, the evidence is based on studies with heterogeneous methodologies. Many of the interventions were delivered as projects to selected groups, raising important questions about the feasibility of wider implementation and the potential for population-wide benefits. Future studies which explore potential effect modifiers and mediators will help to strengthen the evidence base and assess whether interventions have the potential to reduce health inequalities.

## Additional files

Additional file 1: PRISMA 2009 checklist. PRISMA 2009 checklist. (DOC 66 kb)

Additional file 2: Search strategy database(s): Ovid MEDLINE(R) and Ovid OLDMEDLINE(R). Search strategy MEDLINE. (DOCX 14 kb)

Additional file 3: Overview of the health outcomes and scales used to assess the interventions on respect and social inclusion (34 studies in total). Overview of the health outcomes and scales used to assess the interventions on respect and social inclusion. (DOCX 20 kb)

Additional file 4: Summary of the quantitative evidence of the included studies stratified by intervention type. Summary table for quantitative studies [132–135]. (DOCX 94 kb)

Additional file 5: Summary of the qualitative evidence of the included studies stratified by intervention type. Summary table for qualitative studies [136]. (DOCX 42 kb)

Additional file 6: Item-level risk of bias (RoB) assessment for quantitative studies using the Liverpool University Quality Assessment Tool (LQAT) (Pope [61]). Item-level risk of bias assessment for quantitative studies. (DOCX 39 kb)

Additional file 7: Item-level risk of bias (RoB) assessment for qualitative studies using tools adapted from Harden et al. [62] and Mays and Pope [63]. Item-level risk of bias (RoB) assessment for qualitative studies. (DOCX 23 kb)

## Abbreviations

PRISMA: Preferred Reporting Items for Systematic Reviews and Meta-Analyses
WHO: World Health Organization
