# Supplementary material for: What is the impact on health and wellbeing of interventions that foster respect and social inclusion in community-residing older adults? A systematic review of quantitative and qualitative studies
Source: Syst Rev. 2018 Jan 30;7:26. doi: 10.1186/s13643-018-0680-2 (PMC5789687; doi:10.1186/s13643-018-0680-2)
Supplement: Supplementary file 2 — Search strategy database(s): Ovid MEDLINE(R) and Ovid OLDMEDLINE(R). Search strategy MEDLINE. (DOCX 14 kb) [file 13643_2018_680_MOESM2_ESM.docx]

Additional file 2 Search strategy database(s): Ovid MEDLINE(R) and Ovid OLDMEDLINE(R)

| # | Searches |
| --- | --- |
| 1 | dementia/ or alzheimer disease/ |
| 2 | limit 1 to (abstracts and English language and humans and yr="1990 - 2015") |
| 3 | Stroke/ |
| 4 | limit 3 to (abstracts and english language and humans and yr="1990 - 2015") |
| 5 | Depression/ |
| 6 | limit 5 to (abstracts and english language and humans and yr="1990 - 2015") |
| 7 | ("health*" or "disorder*" or "disease*" or "illness" or "mortality" or "morbidity" or "disability" or "depress*" or "dementia" or "mobility" or "ischaemic heart*" or "stroke" or "cerebrovascular accident*" or "falls" or "Alzheimer's" or "Parkinson's" or "psychological distress" or "psychological disorder*" or "psychological symptoms").mp. [mp=title, abstract, original title, name of substance word, subject heading word, keyword heading word, protocol supplementary concept word, rare disease supplementary concept word, unique identifier] |
| 8 | limit 7 to (abstracts and english language and humans and yr="1990 - 2015") |
| 9 | 2 or 4 or 6 or 8 |
| 10 | ("social inclusion" or "community inclusion" or "e-inclusion" or "digital inclusion" or "social cohesion" or "community cohesion" or "neighbo* cohesion" or "social involvement" or "community involvement" or "social integration" or "community integration" or "social engagement" or "civic engagement" or "community engagement" or "intergeneration*" or "social recognition" or "information and communication technolog*" or "social exclusion" or "neighbo* inclusion" or "neighbo* exclusion" or "community participation" or "social participation" or "ageism" or "agism" or "age* stereotyp*" or "age* discrimination" or "digital divide" or "social interaction*" or "social responsabilit*" or "social capital" or "social networks" or "access services" or "access information" or "access opportunit*" or "access facilities" or "access volunteer*" or "access learning" or "social exchange" or "solidarity").mp. [mp=title, abstract, original title, name of substance word, subject heading word, keyword heading word, protocol supplementary concept word, rare disease supplementary concept word, unique identifier] |
| 11 | limit 10 to (abstracts and english language and humans and yr="1990 - 2015") |
| 12 | ("old*" or "elder" or "aged" or "senior*" or "pensioner*" or "ageing" or "aging").mp. [mp=title, abstract, original title, name of substance word, subject heading word, keyword heading word, protocol supplementary concept word, rare disease supplementary concept word, unique identifier] |
| 13 | limit 12 to (abstracts and english language and humans and yr="1990 - 2015") |
| 14 | "aged, 80 and over"/ or frail elderly/ |
| 15 | limit 14 to (abstracts and english language and humans and yr="1990 - 2015") |
| 16 | 13 or 15 |
| 17 | Community Networks/ |
| 18 | limit 17 to (abstracts and english language and humans and yr="1990 - 2015") |
| 19 | Intergenerational Relations/ |
| 20 | limit 19 to (abstracts and english language and humans and yr="1990 - 2015") |
| 21 | 11 or 18 or 20 |
| 22 | 9 and 16 and 21 |
