# Supplementary material for: What is the impact on health and wellbeing of interventions that foster respect and social inclusion in community-residing older adults? A systematic review of quantitative and qualitative studies
Source: Syst Rev. 2018 Jan 30;7:26. doi: 10.1186/s13643-018-0680-2 (PMC5789687; doi:10.1186/s13643-018-0680-2)
Supplement: Supplementary file 3 — Overview of the health outcomes and scales used to assess the interventions on respect and social inclusion (34 studies in total). Overview of the health outcomes and scales used to assess the interventions on respect and social inclusion. (DOCX 20 kb) [file 13643_2018_680_MOESM3_ESM.docx]

Additional file 3 Overview of the health outcomes and scales used to assess the interventions on respect and social inclusion (34 studies in total).

| First author, year, country study, type | Depressive symptoms/  depression | Subjective health/self-rated health/health status | Quality of life | Mental health | Physical health | Wellbeing | Falls | Perceived stress/ anxiety | Other | | |  |
| --- | --- | --- | --- | --- | --- | --- | --- | --- | --- | --- | --- | --- |
| Mentoring interventions | | | | | | | | | | | |  |
| Dickens 2011, UK  QUANT | Geriatric Depression Scale (GDS-10) | Health status (EuroQol EQ-5D) |  | SF-12 –  Mental health component score (MCS) | SF-12 –  Physical health component score (PCS) |  |  |  |  |  | |  |
| Ellis 2004, UK  MIXED |  |  |  |  |  | Short Form 12 Health Survey (SF12) |  |  |  |  | |  |
| Intergenerational interventions (including Ellis 2004) | | | | | | | | | | | |  |
| Chung 2009, China  QUANT | Chinese version of Geriatric Depression Scale (CGDS) |  | Quality of Life-Alzheimer’s Disease (QoL-AD) |  |  |  |  |  |  |  | |  |
| De Souza 2007, Brazil, QUANT |  | Self-rated health  Questions taken from the Brazilian Old Age Scale |  |  |  |  |  |  |  |  | |  |
| Hernandez 2008, Spain, QUANT | Yesavage Depression Scale (YDS) |  |  |  |  |  |  |  |  |  | |  |
| Hong 2010, USA, QUANT | Centre for Epidemiologic Studies Depression Scale (CES-D) | Non-standardised scale |  |  |  |  |  |  |  |  | |  |
| Fujiwara 2009, Japan, QUANT | Short version of Geriatric Depression Scale (GDS) | Non-standardised scale |  |  |  |  |  |  |  |  | |  |
| Gaggioli 2014, Italy, QUANT |  |  | The adapted Italian version of the World Health Organization Quality of Life Scale for Older People (WHOQOL) |  |  |  |  |  |  |  | |  |
| Murayama 2014, Japan, QUANT | Geriatric Depression Scale (GDS) -Short Version-Japanese |  |  |  |  |  |  |  |  |  | |  |
| Fried 2004, USA, QUANT |  |  |  |  |  |  | Non-standardised scale | |  |  | |  |
| Newman 1995, USA, QUANT | Geriatric Depression Scale (GDS) |  |  |  |  |  |  |  |  |  | |  |
| Mendis 1992, USA, QUANT | Center for Epidemiological Studies-Depression Scale (CES-D) |  |  |  |  |  |  |  |  |  | |  |
| Ellis 2004, UK, MIXED |  |  |  |  |  | Short Form 12 Health Survey (SF12) |  |  |  |  | |  |
| Dancing interventions | | | | | | | | | | | |  |
| Houston 2015, UK, QUANT | Dance for Parkinson’s' questionnaire (including the Centre for Epidemiologic Studies Depression Scale) | Dance for Parkinson’s' questionnaire (including questions from SF-36) |  |  |  |  | Non-standardised scale | |  |  | |  |
| Hackney 2007, USA, QUANT | The 17-item Philadelphia  Geriatric Centre Morale Scale |  |  |  |  |  | The modified  Falls Efficacy  Scale | |  |  | |  |
| Music and singing interventions | | | | | | | | | | | |  |
| Coulton 2015, UK, QUANT (same as Clift, 2012) | Hospital Anxiety and Depression Scale (HADS) |  |  | SF-12 –  Mental health component score (MCS) | SF-12 –  Physical health component score (PCS) |  | Hospital Anxiety  and Depression  Scale (HADS) | |  |  | |  |
| Cohen 2006, USA, QUANT | Geriatric Depression Scale–Short Form (GDS) |  |  |  | NS scale |  |  | |  |  | |  |
| Clift 2011, UK  QUANT |  |  |  |  |  |  |  | CORE questionnaire | |  | |  |
| Davidson 2011, Australia MIXED |  |  | Quality of Life – Alzheimer’s Disease (QoL-AD) |  |  |  |  |  |  |  | |  |
| Creech 2013, UK, MIXED |  |  | the Basic Needs Satisfaction Scale  12-item version of CASP |  |  |  |  |  |  |  | |  |
| Davidson 2014, Australia MIXED | Geriatric Depression Scale (GDS) |  |  | Medical Outcomes Study Short-Form (SF-36) Health Survey Version 2 | Medical Outcomes Study Short-Form (SF-36) Health Survey Version 2 |  |  |  |  |  | |  |
| Information-communication technology interventions | | | | | | | | | | | |  |
| Slegers, 2008 the Netherlands, QUANT | 90-item Symptom Check List |  |  |  | 36-item Short-Form  Health Survey (SF-36) | 36-item Short-Form  Health Survey (SF-36) (emotional wellbeing) |  | (Anxiety and sleep complaint)  90-item Symptom Check List |  |  | |  |
| Woodward 2011, USA, QUANT | Geriatric Depression Scale GDS) |  | Non-standardised  scale | |  |  |  |  |  |  | |  |
| Woodward 2012, USA, QUANT | Geriatric Depression Scale GDS) |  | Non-standardised  scale | |  |  |  |  |  |  | |  |
| Art and culture interventions (including Cohen 2006) | | | | | | | | | | | |  |
| Phinney 2014, Canada, MIXED | Geriatric Depression Scale short (GDS short) | Single item perceived overall health scale |  |  |  |  |  | Chronic pain: single  item verbal descriptor scale; Daily function: Older Americans Resources and Services Activities of Daily Living Questionnaire  (OARS-(I)ADL) | | |  |  |
| Cohen 2006, USA, QUANT | Geriatric Depression Scale–Short Form (GDS) |  |  |  | Non-standardised scale |  |  |  |  | |  |  |
| Camic, 2014, UK, MIXED |  |  | Dementia Quality of  Life (DEMQOL-4) |  |  |  |  |  |  | |  |  |
| Yuen 2011, USA, MIXED |  |  |  | 36-Item Short-Form Health Survey (SF-36) | 36-Item Short-Form Health Survey (SF-36) | General Well-being Schedule (GWBS) |  |  |  | |  |  |
| Vogelpoel 2014, UK, MIXED |  |  |  |  |  | Warwick and Edinburgh Mental Wellbeing Scale (WEMWBS); an extension of Thiele and Marsden’s Dynamic Observation scale |  |  |  | |  |  |
| Multi-activity interventions | | | | | | | | | | | |  |
| Saito 2012, Japan, QUANT | Geriatric Depression Scale (GDS) |  |  |  |  | The Life Satisfaction  Index A (LSI-A) |  |  |  |  | |  |
| Greaves 2006, UK MIXED | Geriatric Depression Scale (GDS) |  |  |  | SF12 Health Quality of Life | SF12 Health Quality of Life |  |  |  |  | |  |
| Gonyea 2013, USA, QUANT | Geriatric Depression Scale (GDS)-Short Form of 15 items |  |  |  |  |  |  | 10-item Perceived Stress Scale (PSS) |  |  | |  |
| Kocken 1998, the Netherlands, QUANT |  | Non-standardised scale |  |  |  | Short version of the validated Dutch scale for wellbeing |  |  |  |  | |  |
| Ruffing-Rahal1994, USA, QUANT |  |  |  |  |  | Non-standardised scale |  |  |  |  | |  |
