# Supplementary material for: What is the impact on health and wellbeing of interventions that foster respect and social inclusion in community-residing older adults? A systematic review of quantitative and qualitative studies
Source: Syst Rev. 2018 Jan 30;7:26. doi: 10.1186/s13643-018-0680-2 (PMC5789687; doi:10.1186/s13643-018-0680-2)
Supplement: Supplementary file 4 — Summary of the quantitative evidence of the included studies stratified by intervention type. Summary table for quantitative studies [132–135]. (DOCX 94 kb) [file 13643_2018_680_MOESM4_ESM.docx]

Additional file 4. Summary of the quantitative evidence of the included studies stratified by intervention type.

| **Study no.**  **First author, year published, study type** | | **Country and study design** | **Study participants**  **Recruitment** | **Comparison group(s)** | **Study aim and intervention** | **Outcome measures**  **Scales** | **Summary of main results** | **Risk of bias^1^** |
| --- | --- | --- | --- | --- | --- | --- | --- | --- |
| GROUP N 1: Mentoring interventions | | | | | | | |  |
|  | Dickens 2011 [77], QUANT | UK  Individual controlled trial | Intervention group: N=200 OP  Mean Age: 71.8 years  F>M  Study participants identified from a population of individuals who were in receipt of the intervention | Control group: N=195 OP  Mean Age: 69.8 years F>M  Study participants were receiving usual care through  routinely available health, social and voluntary care services  Study participants recruited through 3 general  practices | To examine the effectiveness of a mentoring programme on the mental health, and physical health of socially isolated OP  Key features:  Study participants were assigned a mentor who worked with them for up to 12 weeks; in rare cases the support was offered for longer. The aim was to build OP’s self-confidence and engage them in social activities. | Mental health status, physical health, health status, depression  SF-12 mental health component score (SF-12MCS); SF-12 physical health component score (SF-12 PCS), health status (EQ-5D), Geriatric Depression Scale (GDS-10) | OP mentoring (intervention group) experienced non-significant improvement in mental health (MD= 0.8; 95% CI -1.5 to 3.2; p = 0.48); physical health (MD= 0.1; p = 0.90); and reduction in depression (MD=0.2; p = 0.29) at 6 months.  In the intervention group, subjective health improved (MD= -0.09;  p < 0.01) over 6 months. However, intervention group participants reported significantly less improvement in subjective health than controls (MD= -0.1; 95% CI -0.1 to -0.03; p < 0.01). | L-M |
|  | Ellis 2004 [65], MIXED (this study is included in the intergenerational interventions as well)  (only QUANT part reported; QUAL excluded for no info on data and analysis) | UK  Before and after one group design | In 2003 N=64 OP  F=M  in 2004 N=39 OP  Almost F=M  Mean age: NR  Study participants were taking part in the mentoring programme | Subjects acted as own controls | To assess the impact of an intergenerational mentoring programme on health and wellbeing of the  Key features:  OP mentored younger  (Y4-6) and older (Y9-11) children by providing learning support (e.g. areas of mathematics), or by taking part in various intergenerational activities | Wellbeing  Short Form 12 Health Survey (SF12) | OP reported non-significant improvement in the physical health questions, mental health scores and in quality of life between the responses for 2002/3 and those recorded for 2003/4 [mean difference and p-values NR in the study] | H |
|  | **GROUP N 2: Intergenerational interventions (including Ellis 2004)** | | | | | | | |
|  | Chung 2009 [68], QUANT | China  Before and after one group design | N= 49 OP with early dementia  Mean age: 79 years  F>M  Study participants recruited from eight-day care centre (convenience sampling)  N=117 youth participants aged between 16 and 25 years | Subjects acted as own controls | To examine the impact of a reminiscence programme, adopting an intergenerational approach, on OP with early dementia and youth volunteers.  Key features:  Each OP was assigned to two youth participants | Quality of life, depression, orientation, mental health, aspects of physical health  Quality of Life-Alzheimer’s Disease (QoL-AD)  Chinese version of Geriatric Depression (CGDS) | OP taking part in the intergenerational programme reported an improvement of 7% in quality of life (MD= -1.91; 95% CI = -3.18, -0.64) and a reduction of 62% in depressive symptoms (MD = 1.86; 95% CI = 0.92, 2.80) within 2 weeks after the completion of the programme (p values NR). | L |
|  | De Souza 2007 [81], QUANT | Brazil  Cluster randomised control trial | Intervention group: N=149 OP  Mean age= 69.5 years  F>M  (only 32 attended the intervention activities)  Follow-up interviews: 134 OP  5 classes randomly allocated to the intervention group | Control group: N= 117 OP  Mean age= 69.5 years  F>M  Follow-up interviews: 103 OP  5 classes randomly allocated to the control group | To see whether participants in an intergenerational reminiscence programme had better scores on self-rated health at the end of the intervention  Key features: intergenerational activities in which OP shared their memories with the students. The intervention was based on the use of reminiscence to promote joint activities | Self-rated health  Questions taken from the Brazilian Old Age Scale | OP taking part in the intergenerational programme (intervention group) experienced non-significant improvement in the self-rated health (p=0.554; OR 1.133: 95% IC 0.749–1.716) at 4 months’ follow-up. | L |
|  | Hernandez 2008 [87], QUANT | Spain  Before and after design with two control groups | Students:  Intervention group N=100  OP: slightly depressed older people  Group 1: N=36 (treatment with students)  Mean age: 74 years  F>M  Group 2: N=35 (treatment with professional)  Mean age: 75 years  F>M  Study participants  took part in weekly recreational activities | Students:  Control group N=79.  Group 3: N=32 (they attended the recreational activities organised in the centre)  Mean age: 75 years  F>M  Study participants  took part in weekly recreational activities | To analyse the effect of an intergenerational programme on OP’s wellbeing  Key features: 32 interactive sessions between students and OP based on service-learning pedagogy. In group 1 the session was led by students; in group 2 by professionals | Depressive symptoms  The Yesavage Depression Scale (YDS) | Group 1 showed a reduction of 26.3% in depressive symptoms scores obtained in the post-treatment evaluation (MD= 3.53 p < .001). Group 2 showed significant a reduction of 8% in depressive symptoms scores obtained in the post-treatment evaluation (MD= 1.11 p < .008). However, this improvement is worse than that shown by Group 1. For Group 3 (control) an increase of the scores obtained in the post treatment evaluation, revealed an increase of depressive symptoms (MD= -2.72  p < .001). | L |
|  | Hong 2010 [82], QUANT | USA  Quasi-experimental two-group pre-post-test design | Intervention group:  N=167 OP  Mean age: 64.78 years  F>M  Study participants were new volunteers taking part in the programme | Control group: N=167 OP  Mean age: 65.14 years  F>M  The control group was established through matching individuals participating in the Health and  Retirement Study (a biennial survey of a large nationally-representative sample of adults over the age of 50) | To evaluate the effects of an intergenerational school programme on OP’s health outcomes over a two-year period  Key features: OP interacted with students in the classroom on a regular basis.  Activities include support students to improve academic achievement | Self-rated health; depression;  Self-rated health: NS  9 items from the Centre for Epidemiologic Studies Depression Scale (CES-D) | OP taking part in the intergenerational programme (intervention group) experienced a decrease in depressive symptoms at 2 years’ follow-up of 18.5% (MD=0.94 p<.001). OP in the intervention group reported non-significant improvement in self-rated health at 2 years’ follow-up (MD=0.03). When adjusting for pre-test health status, socioeconomic status, volunteer history, and boosted propensity scores, the intervention group experienced greater reduction in depressive symptoms (p< .001) at 2 years’ follow-up.  OP in the intervention group experienced greater improvement in self-rated health that were marginally significant (p = .09) at 2 years’ follow-up.  The effect sizes associated with programme participation were 0.73 for depressive symptoms for the intervention group. | L |
|  | Fujiwara 2009 [104], QUANT  (same study as Murayama 2014 [84], QUANT) | Japan  Cluster controlled trial | Intervention group: N=67 OP  (After 12 months n=39 ‘intensive volunteers’ and n=17  ‘volunteers with low frequency’)  Mean age: 68.2 years  F>M  3 cities were selected and study participants were recruited  through newspapers or newsletters  Children recruited from 6 schools, 3 kindergartens, and six child care centres | Control group: N=74 OP  Mean age:  68.7 years  F>M  Study participants recruited from various social activity clubs (e.g. hobby clubs) | To assess the impact of an intergenerational programme on health and depressive symptoms of the participants.  Key features: OP were engaged in reading picture books to children | Self-rated health; depressive symptoms  NS scale; the short version of Geriatric Depression Scale Version Japanese (GDS-S-J) | OP taking part in the intergenerational programme (intervention group) experienced an improvement in self-rated health that was greater among the ‘intensive’ participants of the intervention group (n=37) than the control group (n=60) at 21 months’ follow-up (p < 0.01).  OP (intervention group) experienced a non-significant improvement in self-rated health at 9 months (MD=0.2) when compared to the control group. [Depressive symptoms results reported in Murayama 2014] | M |
|  |  |  |  |  |  |  |  |  |
|  |  |  |  |  |  |  |  |  |
|  | Gaggioli 2014 [75], QUANT | Italy  Before and after one group design | N=32 OP  Mean age: 67.5 years  F/M= NR  Study participants were recruited from different social senior centres  N=114 students  recruited from primary schools located in the same area of OP | Subjects acted as own controls | To test if an intergenerational reminiscence programme could benefit perceived quality of life in OP  Key features:  OP were assigned to 16 groups, each including two OP and six to eight students. A psychologist facilitated the  3 weekly meetings of reminiscing activities (e.g. local traditions, jobs and professions of the past, and historical events) | Perceived quality of life  The adapted Italian version of the World Health Organization Quality of Life Scale for Older People (WHOQOL) | OP taking part in the intergenerational programme experienced an improvement in perceived quality of life of 4.4% (subscale: past, present, and future activity) immediately after the completion of the 3-week programme  (MD= -0.65; p= .05). [further details NR] | M |
|  |  |  |  |  |  |  |  |  |
|  |  |  |  |  |  |  |  |  |
|  | Murayama 2014 [84], QUANT  (same study as Fujiwara 2009 QUANT) | Japan  Cluster controlled trial | Intervention group: N=67 OP (N=26 OP included in the analysis)  Mean age: 68.8 years  F>M  Study participants recruited  through newspapers or newsletters  Study participants who took part in the seminars constituted the intervention group | Control group: N= 82  (N=54 OP included in the analysis)  Mean age: 69.3 years  F>M  Study participants were recruited from hobby clubs, volunteering for adults, etc., but none of them were allowed to engage themselves in intergenerational programs with children | To examine whether participation in the intergenerational programme affected OP’s depressive mood by strengthening their sense of coherence  Key features: OP were engaged in reading picture books to children | Depressive mood  Geriatric Depression Scale-Short Version-Japanese | OP taking part in the intergenerational programme (intervention group) experienced an improved sense of meaningfulness which was positively correlated with depressive mood (p=.001); When controlling for sense of meaningfulness, the overall direct effect of programme participation was reduced  (p < .05). Multiple mediation analysis revealed that participation in the programme was associated with a sense of manageability which was also significantly related to depressive mood.  OP in the intervention group experienced a significant reduction in depression of 14% at time 3 when compared with the control group (MD= 0.31; p < .10). | M |
|  |  |  |  |  |  |  |  |  |
|  | Fried 2004 [76], QUANT  (same study as Rebok 2011 [132] QUANT) | USA  Pilot individual randomised controlled trial | Intervention group: N=70 OP  Mean age: 69 years  F>M  Study participants were recruited through community groups and church in the neighbourhoods around the chosen schools, etc. They were randomly assigned to the intervention or control group.  6 public elementary schools recruited | Control group N= 58  Mean age: 69  F>M  Sampling method for the control group NR | To assess if an intergenerational school programme leads to positive effects on intermediary risk factors for disability and other diseases in OP  Key features: OP worked 15 hours per week. Activities included support literacy development for children, etc. | Falls (fallen in the last 12 months)  NS | OP taking part in the intergenerational programme (intervention group) experienced a non-significant decrease of more than 50% in falls rates (from 15% to 7%) at 4-8 months’ follow-up (p= 0.17) if compared with the controls, wherein falls rate increased from 10% to 13% among the OP. [CIs and p-values NR] | M-H |
|  |  |  |  |  |  |  |  |  |
|  | Newman 1995 [105], QUANT | USA  Before and after one group design | N=26 OP  Mean age: NR  F>M  Convenience sampling | Subjects acted as own controls | To assess the effect of the programme on perceived depression (secondary outcome) in relation to age and educational level (descriptive analysis)  Key features: OP interacted with students in the classroom. OP assisted students in some activities including maths problems, and science experiments. | Perceived depression | OP taking part in the intergenerational programme experienced a reduction in perceived depression of 16.64% at 6-8 weeks’ post-test. Effects by Education level: OP in the lower education group (high school) experienced an increase of in perceived depression 1.61% at 6-8 weeks’ post-test; the higher education group (college) reported a decrease of 26.42% in perceived depression at 6-8 weeks’ post-test. Effects by Age: the older group (70 and over) experienced a decreased in perceived depression of 24.27% at 6-8 weeks’ post-test, while the younger group (60 and over) reported an increase in perceived depression of 4.77% at 6-8 weeks’ post-test. [CIs and p-values NR] | H |
|  | Mendis 1993 [103], QUANT | USA  Non-concurrent multiple baseline design/ before and after one group design | N=20 OP  Mean age: 83.7 years  M/F= NR  Study participants were recruited from a residential facility. OP were randomly assigned to children  N= children: NR | Subjects acted as own controls | To explore the effect that participation in an intergenerational programme may have on the psychological wellbeing of OP  Key features: older people were involved with pre-school children in various activities such as drawings, reading from children's books decorating a cup etc. There were three teachers per class. | Psychological wellbeing; depression  Centre for Epidemiological Studies-Depression Scale (CES-D) | OP taking part in the intergenerational programme experienced a non-significant reduction of 4.2% in depression scores at 8-weeks’ follow-up (MD= -0.97; p=0.3).  [Findings for psychological wellbeing NR] | M-H |
|  | Ellis 2004 [65] , MIXED (this study is included in the intergenerational interventions as well)  (only QUANT part reported; QUAL reported in Table 2) | UK  Before and after one group design | In 2003 N=64 OP  F=M  in 2004 N=39 OP  Almost F=M  Study participants were taking part in the mentoring programme | Subjects acted as own controls | To assess the impact of an intergenerational mentoring programme on health and wellbeing of OP  OP mentored younger  (Y4-6) and older (Y9-11) children by proving learning support (e.g. areas of mathematics), or took part in various intergenerational activities | Wellbeing  Short Form 12 Health Survey (SF12) | OP reported non-significant an improvement in the physical health questions, mental health scores and in quality of life between the responses for 2002/3 and those recorded for 2003/4. [CIs and p-values NR] | H |
| GROUP N 3: Dancing interventions | | | | | | | | |
|  | Houston 2015 [70], MIXED  (only QUANT part reported; QUAL reported in Table 2) | UK  Individual controlled  study | Intervention group: N=24 OP with PD of which the majority was 60 years  M/F= NR  Study participants were volunteers taking part in the programme | Control group: N=15 OP with PD  Sampling method for the control group NR | To examine the effects of a dancing programme for OP with Parkinson’s disease on their health, quality of life, and falls  Key features: OP took part in several dancing sessions led by professionals | Health; quality of life, falls    Dance for Parkinson’s' questionnaire (including questions from SF-36,  UPDRS, Centre for Epidemiologic Studies Depression Scale) | OP taking part in the dancing programme (intervention group) experience non-significant improvements in  subjective health (including change in medication), quality of life, and falls scores between 2 weeks and 6-8 months’ follow-up. [mean difference and p-values NR in the study] | M |
|  | Hackney 2007 [71], QUANT | USA  Individual  Randomised controlled trial | Intervention group (tango): N=9 OP with PD  N=9 without PD of which the majority was 60 years  M/F= NR  Study participants with PD were recruited from the University  School of Medicine’s Movement Disorders Center and the community, and were randomly assigned to one of two groups: tango or traditional exercise. | Control group (traditional exercise);  N=10 with PD  N=10 without PD of which the majority was 60 years | To compare the effects of a 13 weeks’ intervention (tango) to those of traditional exercise on depression and falls in OP with and without Parkinson’s Disease (PD)  Key features: OP took part in several dancing sessions led by professionals | Depression; falls (more confidence in their ability not to fall during daily activities)  The modified Falls Efficacy Scale; The 17-item Philadelphia  Geriatric Centre Morale Scale | Between OP taking part in the tango exercise and those taking part in the traditional exercise there was little reduction in depression scores (OP with PD: MD=0.26; OP without PD: MD= 0.52. P= 0.001) at 10-week follow-up.  OP with PD taking part in the tango dancing programme experienced improvements in the measures of falls (functional reach and one leg stance) at 10-week follow-up if compared to the traditional exercise.  [CIs and p-values NR for falls] | M |
| GROUP N 4: Music and singing interventions | | | | | | | | |
|  | Coulton 2015 [91], QUANT  (same study as  Clift 2012 [133], QUANT) | UK  Pragmatic randomised controlled trial | Intervention group (singing group): N=127 OP  Mean age: 69 years  F>M  Study participants were recruited from  day centres and through local advertisements  and were randomly assigned to the intervention or control group | Control group (non-singing group): N=131 OP  Mean age= 69 years  F>M  Participants of the control group continued with their normal activities | To assess the effectiveness for OP of an engagement in community singing on measures of physical and mental health  Key features:  OP come together to sing with the support of professional musicians | Health-related quality of life; physical health-related components of quality of life; depression and anxiety  York SF-12 mental health  component; SF12 – Physical health component: Hospital Anxiety and Depression Scale (HADS) | OP taking part in the singing programme (intervention group) experienced an improvement of 5% in mental health-related quality of life at 6 months’ follow-up (MD=2.35 p=0.05). However, OP taking part in the singing programme (intervention group) experienced non-significant improvements in the physical health related quality of life (MD 0.26 p=0.73), anxiety (MD= -0.57 p=0.13) and depression (MD= -0.53 p=0.14) at 6 months’ follow-up. At 3 months, OP taking part in the singing programme (intervention group) experienced significant improvements of 9.4% in mental health-related quality of life (MD= 4.77 p <0.01), a reduction of 31.1% in anxiety (MD= −1.78 p <0.01) and of 36.6% in depression (MD=−1.52 p <0.01). | L |
|  | Cohen 2006 [66], QUANT | USA  Individual controlled trial | Intervention group (chorale singing) N=90 OP  F>M  Mean age: 79 years  Study participants were recruited through notices requesting volunteers  Study participants were assigned to either an intervention (chorale) or comparison (usual activity) group. | Comparison group: N=76 OP  Age mean: 79.9 years  The control group was a specific subsample of a longitudinal study | To measure the impact of community-based cultural programmes on the physical health, and mental health of OP  Key features:  The cultural programmes comprised participatory art programmes, ranging from painting, writing, to music in the form of singing in chorales.  The intervention consisted of participating in a  professionally conducted chorale in which there  were weekly singing rehearsals for 30 weeks as well  as public performances | Overall perceived health; falls; depression  Self-reported assessments of general physical health;  Geriatric Depression Scale–Short Form (GDS) | OP taking part in the participatory cultural programme experienced improvements of 9.03% in perceived health (MD= 0.72; p <.01), and a decrease of 104% in fall rates to an average of 0.23 falls per person (p < .05), and fewer other health problems (MD= -0.15 p< .10) than the comparison group at 12 month-follow-up. However, OP taking part in the participatory cultural programme experienced non-significant improvements of 40% in depression  (MD= -0.7) than the comparison group at 12 month-follow-up. | L |
|  |  |  |  |  |  |  |  |  |
|  | Clift 2011 [92], QUANT | UK  A before and after one group design (longitudinal, observational design) | N=137 OP  (N=42 provided sufficiently data on the CORE questionnaire)  Mean age: 59.6 years  F>M  Study participants were volunteers taking part in the programme | Subjects acted as own controls | To describe the development and evaluation of community singing programme with mental health services users  Key features: the new choirs met weekly in  community centres under the direction of facilitators who received training and support as part of the project | Mental distress  CORE questionnaire | OP taking part in the singing programme experienced a reduction of 27.3% in mental distress over a period of 8 months (MD= 2.58 p<0.001) with a moderate effect size of 0.44. Improvements were shown in the three sub-scales of mental distress within the CORE questionnaire: wellbeing (MD=0.37 p< 0.003), problems (MD=0.07 p 0.005) and functioning (MD= 0.29 p< 0.003). The risk sub-scale showed non-significant improvement. | L-M |
|  | Davidson 2014 [94], MIXED  (only QUANT included; QUAL part excluded as data were not stratified by institutional and community setting) | Australia  Pre-and post- mixed methods one group design (the second group was excluded from this review as recruited from an institutionalised setting) | Participants (community group): N=16 OP  Mean age: 70 years  M/F= NR | Subjects acted as own controls | To evaluate the effect of a community singing programme on measures of health and wellbeing in OP  Key features: singing  sessions were led by an experienced community musician at a  local community centre | Physical and mental health; depressive symptoms  Medical Outcomes Study Short-Form (SF-36) Health Survey Version 2; Geriatric Depression Scale (GDS) | OP taking part in the singing programme experienced non-significant reductions in depressive symptoms (MD= -0.2), and in physical and mental health scores. OP taking part in the singing programme experienced a significant improvement of 14.3% in the vitality subscale (MD=10.4; p= 0.03) at 8 weeks’ follow-up. | M |
|  | Creech 2013 [93], QUANT  (same study as Hallam, 2012 [134] MIXED) | UK  Before and after group design with a control group  (3 case studies of musical community involvement and comparisons with control group) | Intervention group: N= 398 OP involved in musical activities  Modal age: 65 years  F>M  Study participants were volunteers taking part in the programme | Comparison group: N= 102  Modal age: 65 years  F>M  OP in the control group participated in non-music activities (e.g. language,  book, yoga, and social groups) | To explore  the impact  of active engagement with music making  on the wellbeing and quality of life of a sample of OP  Key features: musical activities included steel pans, guitars, etc. Participants in each case study site had the opportunity to take part in performances. | Quality of life; subjective wellbeing  The Basic Needs Satisfaction Scale (sub-components control, autonomy and relatedness);  12-item version of CASP to measure the dimensions of control, autonomy, self-realisation and pleasure | OP taking part in the music making programme (intervention group) experienced improvements in two components of the quality of life scale. An increase of 14% in control; mean difference 1.15; p=.0001; increase of 7.6% in pleasure; mean difference 0.8; p=.0001 at 9 months’ follow-up if compared to the controls, but non-significant improvements in the other two components of the quality of life scale related to autonomy or self-realisation.  OP taking part in the music making programme (intervention group) experienced improvements in the total score of the subjective wellbeing scale (p = .01), and in the sub-scale linked to relatedness (p = .002) at 9 months’ follow-up. | M |
| 19 | Davidson 2011 [69], MIXED  (only QUANT included; QUAL part excluded as data were not stratified by institutional and community setting) | Australia  Before and after mixed methods one group design (the second group was excluded from this review as recruited from an institutionalised setting) | Participants (community group): N=23 including 11 OP with dementia  Mean age: 71 years  M/F=NR  N=11 caregivers  Study participants were recruited from a community centre | Subjects acted as own controls | To assess the impact of the singing programme on the quality of life of OP  Key features: 6-week singing program targeted for OP with dementia and their caregivers and delivered by a program facilitator | Quality of Life – Alzheimer’s Disease (QoL-AD) | OP taking part in the singing group experienced non-significant improvements in the overall quality of life scores, or for scores given to each individual item at 6 weeks’ follow-up. This is in contrast with what observed by combining the two groups of OP and by their carers. [Further details on results NR] | H |
|  |  |  |  |  |  |  |  |  |
| GROUP N 5: Information-communication technology interventions | | | | | | | | |
| 20 | Slegers 2008 [67], QUANT (same study as Slegers 2013 [135] QUANT) | Netherlands  Individual randomised controlled trial | Randomisation 1  Intervention group: N=123 OP (training)  aged between 64 and 75 years  M/F=NR  Randomisation 2  Group 1 (training and intervention): N=62 OP  Group 2 (training– no Intervention): N=61 OP  Study participants were recruited through flyers  and were randomly assigned to the intervention or control group | Randomisation 1  Group 3 (no training–no Intervention): N=68 OP  Group 4:  (participants with no interest in computer use): N=45 OP | To examine the impact of a computer training programme on between the measures of physical wellbeing, social wellbeing, and emotional wellbeing in OP  Key features: OP in in the intervention group received a personal computer with a broadband Internet connection and were given weekly and monthly Internet assignments and a helpdesk | Physical health; emotional wellbeing (depression, anxiety, and sleep complaints)  36-item Short-Form  Health Survey (SF-36),  90-item Symptom Check List  SCL Anxiety  Self-report measures of wellbeing and quality of life | OP taking part in the computer training programme (Group 1 training and intervention) experienced 5% non-significant improvements on the physical health (MD=2.63; p=0.14) and mental health (MD=1,03; p=0.10). Group 1 showed also a non-significant reduction in depression (MD=-1.4; p=0.56), anxiety (MD= -.025; p=0.13), and sleep complaints (p= 0.89) at 4 and 12 months’ follow-up when compared with the other groups (training-no intervention; no training-no intervention; not interested) | L |
| 21 | Woodward 2011 [95], QUANT | USA  Individual randomised controlled trial | Intervention group: N=45 OP (first wave Technology and Aging Programme (TAP1))  Mean age: 72 years  F>M  Study participants were survey respondents  who expressed interest in participating in future research  and were randomly assigned to the intervention or control group | Control group  N=38 OP (first wave of TAP1)  Mean age: 72 years  F>M  Participants who expressed an interest in participating in the project | To examine the impact of a computer training programme on OP’s mental health related outcomes  Key features: classes were taught by a project coordinator and topics included basics of using a computer, using voice and video via the Internet, etc. every 2 weeks. | Mental health related outcomes (depression; quality of life)  NS scale for quality of life; Geriatric Depression Scale (GDS) | OP taking part in the computer training programme experienced improvements in quality of life at 6 months’ follow-up (4.99 increase on a 16-112 scale; p<.05) and non-significant reduction in depressive symptoms (-0.12 decrease on a 0-15 scale; p value not reported) at 6 months’ follow-up if compared to the control group. | M |
| 22 | Woodward 2012 [78], QUANT | USA  Before and after group study one controlled group design | Intervention group (second wave Technology and Aging Programme (TAP2)): N= 19 OP  Mean age: 73 years  F>M  Participants were from the control group of a previous programme (TAP1) [see above Woodward 2011] | Control group (first wave Technology and Aging Programme (TAP1)): NR (N=45) OP  Mean age: 72 years  F>M  This study used as a control group a sample of participants who participated in a previous programme TAP1 [see above Woodward 2011] | To test a peer tutor model to teach older adults how to use information and communication technologies, and to examine its impact on mental health related outcomes  Key features:  weekly programme based on a peer tutor model to teach OP how to use information and communication technologies | Mental health related outcomes (depression; quality of life)  NS scale for quality of life; Geriatric Depression Scale (GDS) | OP taking part in the peer tutor model computer training programme (TAP2) experienced non-significant improvements in quality of life (6.1 increase on a 16-112 scale; p value not provided) and depressive symptoms (0.2 increase on a 0-15 scale; p value not provided) at 3, 6 and 9 months’ follow-up if compared with the control group (TAP1) staff-directed model. | M-H |
| GROUP N 6: art and culture-based interventions (including Cohen 2006) | | | | | | | | |
|  | Phinney 2014 [96], MIXED  (only QUANT part reported; QUAL reported in Table 2) | Canada  Before and after one group design | N=51 OP (4 groups)  F>M  Mean age: 73.5 years  Recruitment occurred through the Alzheimer’s Society, Extra Care Charitable Trust and  the host galleries | Subjects acted as own controls | To evaluate the effect of a community-engaged arts programme on the physical, emotional, and social wellbeing of OP  Key features: Weekly workshops for 3 years where artists  worked with OP to produce a collective art piece or performance for public presentation | 2 domains of health: physical wellbeing (daily function, perceived health status, chronic pain); emotional wellbeing (depressive symptoms)  PHYSICAL wellbeing:  Older Americans Resources and Services Activities of Daily Living Questionnaire  (OARS-(I)ADL); single item perceived overall health; single item verbal descriptor scale  EMOTIONAL wellbeing: Geriatric Depression Scale short (GDS short) | OP taking part in the community-engaged arts programme experienced improvements of 14% in perceived health (MD= -0.4; p< .10; medium effect size d= 0.41) and a reduction of 23 % in chronic pain measures (MD=0.5 p<.05; medium effect size d=0.52) at 2 year and half follow-up. OP also experienced non-significant improvements in daily function (MD=0.4, effect size d=0.19) and reduction of 22% in depression measures (MD= 0.7, effect size d=0.20) at 2 year and half follow-up | L-M |
|  | Cohen 2006 [66], QUANT | USA  Individual controlled trial | Intervention group (chorale singing) N=90 OP  F>M  Mean age: 79 years  Study participants were recruited through notices requesting volunteers  Study participants were assigned to either an intervention (chorale) or comparison (usual activity) group. | Comparison group: N=76 OP  Age mean: 79.9 years  The control group was a specific subsample of a longitudinal study | To measure the impact of community-based cultural programmes on the physical health, and mental health of OP  Key features: the cultural programmes comprised participatory art programmes, ranging from painting, writing, to music in the form of singing in chorales.  The intervention consisted of participating in a  professionally conducted chorale in which there  were weekly singing rehearsals for 30 weeks as well  as public performances | Overall perceived health; falls; depression  Self-reported assessments of general physical health;  Geriatric Depression Scale–Short Form (GDS) | OP taking part in the participatory cultural programme experienced improvements of 9% in perceived health (MD= 0.72; p <.01), and a decrease of 104% in fall rates to an average of 0.23 falls per person (p < .05), and fewer other health problems (MD= -0.15 p< .10) than the comparison group at 12 month-follow-up. However, OP taking part in the participatory cultural programme experienced non-significant improvements of 40% in depression  (MD= -0.7) than the comparison group at 12 month-follow-up. | L |
|  | Camic 2014 [97], MIXED  (only QUANT part reported; QUAL reported in Table 2) | UK  Before and after one group design (2 intervention sites) | N=24 OP (12 with dementia)  Mean age=78.3 years  M/F=NR  Study participants were recruited  through the  Alzheimer’s Society, Extra Care Charitable Trust and the host galleries | Subjects acted as own controls | To examine the impact on OP’s quality of life of an 8-week art-gallery-based intervention conducted at two distinct types of galleries (traditional and contemporary)  Key features:  sessions included one hour of art viewing and discussion followed by one hour of art making | Health-related quality-of-life  Dementia Quality of  Life (DEMQOL-4) questionnaire | OP taking part in both art-gallery-based interventions (traditional and contemporary) groups experienced non-significant improvements in health-related quality of life (p=0.88) [Further details on results NR]. | M |
|  |  |  |  |  |  |  |  |  |
|  | Yuen 2011 [98], MIXED  (only QUANT part reported; QUAL reported in Table 2) | USA  Before and after one group design | N=12 OP  Aged between  62–88 years’ old  F>M  Study participants were volunteers currently involved with the programme | Subjects acted as own controls | To evaluate the impact of the theatre programme on OP’s psychological wellbeing and health-related quality of life  Key features: the theatre programme included a 6-week acting class and 4 public performances | Subjective wellbeing; distress; health-related quality of life  General Wellbeing Schedule (GWBS); the 36-Item Short-Form Health Survey (SF-36) | OP taking part in the theatre programme experienced improvements of 27.6% in subjective wellbeing (MD=-20.2, p=.002), and of 21.1% in physical health (MD= -11.9, p=.030) at one-month follow-up. OP taking part in the theatre programme experienced non-significant improvements of 3.7% in mental health (MD= -2.8; p=.154) at one-month follow-up. | M |
|  | Vogelpoel 2014 [74], MIXED  (only QUANT part reported; QUAL reported in Table 2) | UK  Before and after one group study design | N=12 OP with sensory impairments  (measures of wellbeing collected for N= 8)  Mean age: 80 years  F>M  Study participants were referred by general practices | Subjects acted as own controls | To describe the benefits on wellbeing of a 12-week social prescribing art service programme for OP with sensory impairments experiencing social isolation  Key features:  The programme included participation in an arts workshop programme; ongoing individual assessments of health status; and ongoing observations of OP’s health status | Wellbeing  Warwick and Edinburgh Mental Wellbeing Scale (WEMWBS); an extension of Thiele and Marsden’s Dynamic Observation scale | OP taking part in the social prescribing service programme experienced non-significant improvements of 14.6% in wellbeing at the last week of the programme (MD=-6) [Further details on results NR]. | M-H |
|  |  |  |  |  |  |  |  |  |
| GROUP N 7: Multi-activity interventions | | | | | | | | |
|  | Saito 2012 [99], Japan, QUANT | Individual randomised controlled trial | Intervention group:  N=21 socially isolated OP aged between 66–84 years  F>M  Study participants were recruited from the  Basic Resident Registration Cards  Participants were randomly allocated to the intervention or control group | Control group: N=42 OP aged between 66–84 years  F>M  OP in the control group were sent several newsletters or written information about group activities in the city during the intervention period | To examine the effect of a programme focused on preventing social isolation on depression, and subjective wellbeing of OP  Key features: four sessions of a group-based programme were designed to prevent social isolation by improving community knowledge and networking with other participants and community gatekeepers | Depression; subjective wellbeing  Geriatric Depression Scale (GDS); The Life Satisfaction  Index A (LSI-A) | OP taking part in the programme experienced (intervention group) improvements of 9% in subjective wellbeing  (p =0.039) at 6 months’ follow-up (MD= 1.9).  OP in the intervention group experienced a non-significant reduction in depression at 1 and 6 months’ follow-up (MD= 1.1 at 1 month. MD= 0.4 at 6 months) [p-values for depression NR]. | L-M |
|  | Greaves 2006 [100], MIXED  (only QUANT part reported; QUAL reported in Table 2) | UK  before and after one group design | N=229 socially isolated OP  Mean age=77 years  F>M  (Data were available for N=172 OP at baseline, N= 72 OP at 6 months and N=51 OP  at 12 months)  Study participants purposively selected among those who participated in the programme | Subjects acted as own controls | To evaluate the impact of a programme focused on preventing social isolation on the depressive symptoms, and on physical and mental wellbeing of OP  Key features: activity-based interventions were combined with visits from peers initially on a weekly basis, and regular telephone contact, which is gradually diminished as participants become more confident  Participants determined programmes of creative, exercise and/or cultural activities | Depressive symptoms; physical and mental wellbeing/health  Geriatric Depression Scale (GDS)  SF12 Health Quality of Life | OP taking part in the programme experienced improvements of 6.4% in mental health (MD=3, 95% CI: 1.01-5.04, p < 0.005)  and a reduction of 13.4% in depressive mood (MD = 0.60, 95% CI: 0.14-1.05, p <0.02) at 6 months’ follow-up.  OP taking part in the programme experienced non-significant improvements in physical health (p=0.996) at 6 months’ follow-up.  At 12 months, OP taking part in the programme experienced non-significant improvements in mental health (p=0.654), and improvements of 11.6% in depressive mood (MD =0.56, 95% CI: 0.02-1.11. p < 0.05). Physical health was close to significance (MD = 1.57,  95% CI: –0.08-3.22; p= 0.06).  OP taking part in the programme experienced improvements in health utility scores (combining mental and physical components) at 12 months’ follow-up (MD= 0.027, 95% CI: 0.002-0.052, p < 0.05). | M |
|  | Gonyea 2013 [107], QUANT | USA  Before and after one group design | N=33 OP  Mean age: 81 years  M/F=NR  Study participants were recruited from the census data | Subjects acted as own controls | To examine a support community based programme and the effects on stress, and depression on OP  Key features: the programme was a neighbourhood-based membership organisation supported OP’s ability to continue to live in their homes even as their functional abilities decline. Activities included regular gatherings in neighbours’ homes, meetings, special events, and a weekly personal telephone call from a fellow member. | Perceived stress; depressive symptoms  10-item Perceived Stress Scale (PSS); Geriatric Depression Scale (GDS)-Short Form of 15 items | OP taking part in the community based programme experienced 11.7% reductions in perceived stress (MD=2.23 p < .001), and non-significant reductions in depression at 9 months’ follow-up (MD=0.03 p > .33). [At enrolment, the OP’s mean for depression was 3.64; although no individual scored 10 or higher (indicative of severe depression), 18% were in the 6 to 9 range, suggestive of at least mild depression] | L-M |
|  | Kocken 1998 [79], QUANT | Netherlands  Individual controlled trial | Intervention group: N=138 OP of which the majority was aged 65 years  F>M  Study participants were volunteers interested in taking part in the programme | Control group:  N=182 OP of which the majority was aged 65 years  F>M  Controls were people in the waiting list to join the programme | To evaluate the effect of participation in a health promotion programme on the wellbeing and subjective health of OP  Key features: the course included social clubs, exercise programmes and memory training. It was facilitated by peers, called senior health educators, who had received prior to the course intensive training | Wellbeing; subjective health  Short version of the validated Dutch scale for wellbeing; NS scale or subjective health | OP taking part in the health promotion programme (intervention group) experienced improvements of 5.15% in subjective health immediately after the programme and at 3 months’ follow-up, when compared to the control group (MD=0.37; p < 0.01 99% CI 0.02–0.72).  OP in the intervention group experienced non-significant improvements in wellbeing at 3 months’ follow-up (MD= 0.42 95% CI 20.43–1.28). | L-M |
|  |  |  |  |  |  |  |  |  |
|  |  |  |  |  |  |  |  |  |
|  | Ruffing-Rahal 1994 [73], QUANT | USA  Individual controlled trial | Intervention group: N=14 OP  recruited purposively  M/F= F only  Study participants were recruited purposively for participation in the intervention group | Control group: N=14 OP  A comparable but non-equivalent control group of people living in the same metropolitan census area was followed over the same time | To assess the impact of a 26 weeks’ health promotion programme on the wellbeing of OP  Key features: weekly meetings where group facilitators encouraged OP to discuss health information and self-care topics including their feelings and health | Psychological-spiritual wellbeing  The integration inventory as a measure of psychological-spiritual wellbeing | OP taking part in the health promotion programme (intervention group) experienced non-significant improvements in psychological-spiritual wellbeing scores at 6 months’ follow-up when compared to the control group (MD =  1.47; p 0.2699). | M-H |

Legend 4 *NA= not applicable; NS= non-standardised; NR= not reported; OP= older people (aged 60+ or where the age mean/mode/median is 60+); CI= confidence interval; N= number; Risk of bias: H= high; M= medium; L= low; QUANT= quantitative; MIXED= mixed methods; MD= mean difference; M= mean; 1 Liverpool Quality Assessment Tools (Pope [61]).*
