# Supplementary material for: What is the impact on health and wellbeing of interventions that foster respect and social inclusion in community-residing older adults? A systematic review of quantitative and qualitative studies
Source: Syst Rev. 2018 Jan 30;7:26. doi: 10.1186/s13643-018-0680-2 (PMC5789687; doi:10.1186/s13643-018-0680-2)
Supplement: Supplementary file 5 — Summary of the qualitative evidence of the included studies stratified by intervention type. Summary table for qualitative studies [136]. (DOCX 42 kb) [file 13643_2018_680_MOESM5_ESM.docx]

Additional file 5. Summary of the qualitative evidence of the studies included in the systematic review stratified by intervention type.

| **First author, year published, study type** | | **Country and study design**  **Methods** | | **Study participants**  **Recruitment** | **Aim of the study relevant for this review** | **Mediating factors/mechanisms identified by OP as important in improving health and wellbeing** | **Summary of main results** | **Risk of bias^2^** |
| --- | --- | --- | --- | --- | --- | --- | --- | --- |
| Mentoring interventions | | | | | | | |  |
|  | Ellis 2003 [85], MIXED  (only QUAL part reported; QUANT excluded for no info on data and analysis) | UK  Qualitative study  Focus group discussions (N=9) | | N=42 OP, of which majority were aged 60-75 years  F/M= NR  Study participants were volunteers currently involved with the programme  N=54 students (11-12 years old) | To investigate the use, relevance, and effectiveness of a mentoring programme, adopting an intergeneration approach, targeting secondary school children in need of support and guidance from older mentor volunteers | Making a difference (feeling valued): mentors reported development of positive relationships with children (quotes provided).  Improved confidence, self-esteem and happiness: mentors reported how being involved in the programme increased their confidence and self-esteem by seeing how mentees improved (quotes provided).  Coping: Mentors found the programme helpful as a coping strategy for retirement (quotes provided) | OP reported an enhanced physical and mental wellbeing as a result of taking part in the intergenerational mentoring programme. Being a mentor and being involved in the programme helped OP going through difficult times in their lives. | M-H |
| Intergenerational interventions (including Ellis 2003) | | | | | | | | |
|  | De Souza, 2003 [86], QUAL | Brazil  Qualitative study (focus groups) | | N=26 OP were divided into 3 groups separated by sex.  Aged 60 and over  1 M group and 2 F groups  F>M  Study participants were volunteers who have to have been participating in the programme for at least a year  N=84 students (13-19 years old)  randomly selected and divided into  9 groups | To evaluate the intergenerational programme from the participants’ viewpoint and assess the impact on the health and wellbeing  Key features: the programme aimed at improvising social cohesion, self-perception of OP health status, reduce stereotypes between generations, and enhance social capital. 10 sessions comprised of five groups with 40 students and 5 OP each. Reminiscence activities included discussing old objects. OP were encouraged to share their experiences with the students over the course of a year. | Female groups reported an improvement in perceptions of ageing and towards the younger generation (quotes provided); improved interactions with young people.  Female groups reported reduced isolation and sharing their emotions; enjoying partying together (quotes provided). All groups reported strengthening sympathies and developing empathy (quotes provided).  Male groups reported satisfaction with being admired by young people, the opportunity of meeting other people and taking part in other activities as well as a feeling of freedom (quotes provided) | Female and male groups reported improvement in their health status. E.g. related relief of their pain and aches (quotes provided). All female groups reported that the intergenerational programme helped them to alleviate their depressive mood and to improve their overall wellbeing and humour (quotes provided) | L-M |
|  | Weintraub 2007 [89], QUAL  (same study as Weintraub 2009 [136], QUAL) | USA  Qualitative study (guided interviews) | | N=13 OP of which majority were aged 77-83 years  F>M  Aged 65-90 years’ old  Study participants were volunteers recruited through fliers and room visit invitations  N=7 OP (contact group: occasional interactions with children); N=6 OP (engaged group: numerous interactions with children and participated regularly in the programme)  Children attended the child care centre.  Sample and sampling method NR | To examine perceptions held by OP about the impact of the programme on their emotional and physical wellbeing.  Key features: the centre has an OP day program and a child care centre serving preschool age children in a single building and provides opportunities for daily interaction between children and OP.  Two respondents had daily contact with their children whereas others saw their children anywhere from twice a month to two times a year. | Emotional wellbeing: OP in the engaged group reported more positive attitudes regarding the children and their involvement than did participants in the contact group.  Dimensions of emotional wellbeing: responses of those in the contact group suggested that they were impacted through processes related to peer support, the freedom of choice, manners, and youth and enthusiasm as important dimensions of emotional wellbeing.  The engaged group identified being needed. OP in both groups mentioned the importance of familial connections. OP from the contact group reported an increased physical movement. They spoke of the necessity to be more physically active around the children. OP in the engagement group reported engaging in more physical activity by virtue of lifting and carrying children during intergenerational activities. | Emotional wellbeing: OP reported a positive impact on emotional wellbeing. Seven themes were identified all contributing to the emotional wellbeing (quotes provided). OP reported a feeling of calm as a result of being surrounded and interacting with children (quotes provided).  Physical wellbeing: OP in the engaged and contact group reported physical benefits on wellbeing. Six themes were identified all contributing to the physical wellbeing (quotes provided). OP in the contact and engagement groups reported feeling that existing physical limitations reduced their participation in the programme. OP reported a sense of accomplishment that left them feeling good long after they went home for the day. Attending the programme made OP feeling better, and it was perceived as a recovery strategy. | L-M |
|  | Ellis 2003 [85], MIXED  (only QUAL part reported; QUANT excluded for no info on data and analysis) | UK  Qualitative study  Focus group discussions (N=9) | | N=42 OP, of which majority were aged 60- 75 years  F/M=NR  Study participants were volunteers currently involved with the programme  N=54 students (11-12 years old) | To investigate the use, relevance, and effectiveness of a mentoring programme, adopting an intergeneration approach, targeting secondary school children in need of support and guidance from older mentor volunteers  All the sample schools were involved with the scheme for a minimum of three years. | Making a difference (feeling valued): mentors reported development of positive relationships with children (quotes provided).  Improved confidence, self-esteem and happiness: mentors reported how being involved in the programme increased their confidence and self-esteem by seeing how mentees improved (quotes provided).  Coping: Mentors found the programme helpful as a coping strategy for retirement (quotes provided). | OP reported an enhanced physical and mental wellbeing as a result of taking part in the intergenerational mentoring programme. Being a mentor and being involved in the programme helped OP going through difficult times in their lives. | M-H |
| Dancing interventions | | | | | | | | |
|  | Houston 2011 [72], MIXED  (only QUAL part reported; QUANT part excluded) | UK  Qualitative study  (Pre, during, and post-test semi-structured interviews.  Diaries written by participants of their lives during the programme, or their perceptions of each session) | N=24 OP with Parkinson’s disease, of which majority were aged 60 and over (N=14 chose to be interviewed, N=4 kept diaries; observations of N=24)  F/M=NR  Study participants were volunteers currently involved with the programme | | To explore the impact on health and OP’s experiences from 12 dance sessions  Key features: the programme introduced OP to the ballet *Romeo & Juliet* and provided 12 dance sessions of structured and creative movement accompanied by live piano and flute | Other outcomes reported by participants included (quotes provided): Fluency and dynamics of movement and mobility, loosening of the spinal area, Balance and stability, posture, energy, body awareness, determination and achievement, freedom, sociability, dancing as a group, aid to daily life, confidence, and learning | N=2 OP reported an impact on subjective health (quotes provided). Particularly, they mentioned of their recent visits to their consultants, which occurred during the last few weeks of the dance programme. OP talked about how the programme made them feel better, giving them a sense of wellbeing (quotes provided) | M-H |
|  | Houston 2015 [70], MIXED  (only QUAL part reported; QUANT reported in Table 2) | UK  Qualitative study  (Multiple interviews, focus groups and  observations over the course of three years) | | N=43 OP with Parkinson’s of which the majority was aged between 60 and 80 years old.  F/M=NR  Study participants were volunteers currently involved with the programme | To examine the effects of the dancing programme on OP’s health, quality of life, and falls over the course of 3 years  Key features: introduced OP to various ballets and provided dance sessions of structured and creative movement accompanied by live piano and flute | Other outcomes reported by participants included (quotes provided): fluency of movement and mobility, balance and stability, posture, cognitive functioning, aid for life, relationships, motivation (e.g. feeling included), freedom, social participation | OP reported a positive impact on physical health (quotes provided). Several described the general feeling of being energised, rather than more specific physical changes (quotes provided). OP reported how the programme contributed to their sense of wellbeing (quotes provided), and to feeling good and capable despite some worsening of symptoms (quotes provided) | M-H |
| Music and singing interventions | | | | | | | | |
|  | VarVarigou 2012 [101], QUAL | UK  Qualitative study (qualitative comments offered in response to open questions included in the questionnaires. comments  are combined with data from pre-post-test face-to-face interviews) | | N=27 OP  Mean age: 69 years  F>M  Study participants were selected randomly from a larger group of questionnaire respondents who had expressed an interest in being interviewed and had provided  their contact details | To explore the benefits from the programme based on active music-making reported by OP who participated in weekly musical activities led by professionals over the period of one year | OP reported cognitive benefits (quotes provided): rising to new challenges, acquiring new skills, improved concentration and memory and a general sense of achievement. Social benefits (quotes provided): sense of belonging, opportunities to socialise, a sense of playing a valued and vital role within a community, having fun and having contact with younger people in intergenerational groups.  Emotional benefits and mental health (quotes provided): positive feelings, sense of purpose, renewed sense of vitality and rejuvenation and improved mobility, enhanced confidence, positive feelings about life in general, and support following bereavement | OP reported: improvements in quality of life, cognitive, social, emotional, and physical benefits as a result of taking part in the programme. OP described emotional and mental health benefits: ability to cope effectively with stress, and protection against depression. Although OP reported that health constraints (e.g. hearing) sometimes made participation in and enjoyment of music difficult, they reported improvements to physical health as a result of the programme (e.g. good for asthma and breathing) | L-M |
|  | Skingley 2010 [102], QUAL | UK  Qualitative study  Interviews | | N=17 OP  Mean age: 77 years  F>M  Study participants were recruited from six clubs (of twelve then existing) | To examine OP’s experiences of the singing programme, particularly their potential benefits on health and wellbeing  Key features: community-based groups providing opportunities for OP to  come together and sing, facilitated by experienced musicians  and volunteers | Other outcomes reported by OP included: enjoyment (quotes provided); increased social interactions (quotes provided); cognitive stimulation and learning (quotes provided); and improved memory and recall (quotes provided) | OP reported better mental health (e.g. breathing) and wellbeing (quotes provided) and improvements in physical health (quotes provided) | M |
| Information-communication technology (ICT) interventions | | | | | | | | |
|  | Schlag 2011 [80], QUAL | USA  Qualitative study (two in depth interviews with each participant) | | N=7 OP aged between 64-81 years’ old  F>M  Study participants were recruited from a pool of OP who completed the questionnaires and who were chosen for this study | To examine the effects of computer technology-use and involvement on the lives of OP who participated in the information-communication technology programme.  Key features: the programme relied on peer volunteers to teach and coach | OP reported that ICT use helped them to improve their health maintenance (e.g. researching health issues).  OP related their enhanced sense of wellbeing acquired from the ICT use to an increased sense of purpose, accomplishment, productivity, usefulness, and enjoyment to their lives. Some OP reported the programme served as a medium for strengthening existing relationships. Several OP mentioned that having ICTs as a common interest brought them closer to family members, and mentioned using ICT to keep in touch with family members. For several OP the training undertaken in the programme was a means of enhancing their effectiveness in other volunteer pursuits (e.g. sharing their ICT knowledge with others) | OP reported enhanced sense of wellbeing because of taking part in the programme | M |
| Art and culture interventions | | | | | | | | |
|  | Phinney 2014 [96], MIXED  (only QUAL part reported; QUANT reported in Table 2) | Canada  Qualitative study  (focus groups interviews) | | N=51 OP  Four groups of OP aged between  55 to 90 years Mean age: over 60 years  F>M  Study participants were purposively recruited from diverse range  of urban neighbourhoods and communities | To evaluate the effect of the programme on the physical, emotional, and social wellbeing of OP  Key features: weekly workshops over a three-year period at community centres where artists worked with four groups of OP to produce a collective art piece or performance for public presentation | OP reported an enhanced sense of cohesion and commitment among themselves, describing a “feeling of belonging” and having a place “within community” (quotes provided). OP felt a strong sense of belonging and commitment within the group, and felt more socially connected beyond the group as well. OP reported that their involvement in the programme enhanced their status as valued members of society (quotes provided) | OP reported that the programme helped them to sustain a healthy lifestyle and to overcome some health barriers including depression and chronic health problems, and as a result they felt more socially and physically active (quotes provided).  OP also described how creative work was an opportunity to focus their attention on something beyond themselves, and reducing their feelings of stress and anxiety (quotes provided). | L-M |
|  | Yuen 2011 [98], MIXED  (only QUAL part reported; QUANT reported in Table 2) | USA  Qualitative study (semi-structured interviews) | | N=12 OP aged between  62–88 years  F>M  Study participants were volunteers currently involved with the programme | To evaluate the impact of the programme on OP’s psychological wellbeing and health-related quality of life  Key features: the theatre programme included a 6-week acting class and four public performances | OP reported some factors relating to psychological wellbeing and health-related quality of life: improved self-worth (quotes provided), and self-advocacy (quotes provided), overcoming self-imposed limitations (quotes provided)  Self- worth: several OP were surprised that they could do or learn something new that they did not think they could. As a result of this achievement, it heightened their sense of self-worthiness. Self-advocacy: some OP reported that they had their confidence boosted after the programme. Overcoming self-imposed limitations: Some OP reported that taking part in the programme made them push themselves to stay more active and focussing less on their health problems. | OP who participated in the programme revealed an improvement in their psychological wellbeing and health related quality of life. | L-M |
|  | Vogelpoel 2014 [74], MIXED  (only QUAL part reported; QUANT reported in Table 2) | UK  Qualitative study (semi-structured interviews) | | N=12 OP with sensory impairments aged between 61 to 95 years  Mean age: over 80 years  F>M  Study participants were referred by general practices | To describe the benefits on wellbeing of a social prescribing art service for OP with sensory impairments experiencing social isolation  Key features: participation in a 12-week arts workshop programme; ongoing individual assessments of health status; and ongoing observations of participant’s health statuses | OP reported increased self-confidence through involvement in different art-making processes. The programme enabled OP to build relationships and new friendships (quotes provided); OP experienced: reduced social isolation (quotes provided); sense of belonging and group cohesion (quotes provided); enjoyment in engaging with art or craft (quotes provided) | Mental wellbeing: some OP reported an improvement in their psychological wellbeing (quotes provided). They also reported reduction in perceived stress: the biggest positive change reported by OP was “feeling more relaxed”. | H |
| Multi-activity interventions | | | | | | | | |
|  | Buijs 2003 [90], QUAL | Canada  Qualitative study (semi structured interviews) | | N=23 OP aged between 61 to 90 years old.  Mean age: 76 years  F>M  Study participants were volunteers who participated in the programme.  N=4 OP  Aged between 74 to 84 years old. Mean age: 77 years  F>M  Study participants recruited among the group of programme withdrawers | To assess the impact of a 10-month health promotion programme on OP’s health and wellbeing  Key features: programme interventions delivered in 7 OP’s apartment buildings included exercise classes, health information sessions (*i.e*. health corners), and newsletters | Specific impacts were also noted in physical, mental, and social domains. Five key program processes that linked program interventions and impacts were: having fun, adapting program delivery, providing opportunities for autonomy, encouraging social interactions, and developing meaningful staff–participant relationships (quotes provided).  Barriers to participation, reported by participants, were other priorities, deteriorating health, and forgetting to come. | The main impact reported by OP was ‘feeling better’ (quotes provided). Specific physical health impacts described were being more flexible, having increased strength, noting some weight loss, and experiencing less pain | L-M |
|  |  |  | |  |  |  |  |  |
|  | Greaves 2006 [100], MIXED  (only QUAL part reported; QUANT reported in Table 2) | UK  Qualitative study (semi-structured interviews and one focus group) | | N=18 OP  F>M  Age range: NR  Study participants purposively selected among those who participated in the programme (one additional focus group was conducted with further 8 participants) | To evaluate the impact of the programme on the depressive symptoms, and on physical and mental wellbeing of OP  Key features: Activity-based interventions were combined with visits from peers initially on a weekly basis, and regular telephone contact, which is gradually diminished as participants become more confident and able.  Participants determined programmes of creative, exercise and/or cultural activities | The data indicated a wide range of responses (both physical and emotional), including increased alertness, social activity, self-worth, optimism about life, and positive changes in health behaviour. Psychological and social benefits: The vast majority of OP reported increased confidence in engaging in new activities, and in interacting socially with others. Physical health benefits: four of the 18 OP provided of changes affecting multiple aspects of their lives: increased sense of meaning in life, increased social and physical activity, and more attention to self-care (quotes provided) | OP reported psychological and physical health benefits (quotes provided). Only 3 of the 18 individually interviewed OP reported no change in their mood or health-related behaviours since they became involved with the programme. Physiological and physical health benefits include recovery from depression and better quality of sleep (quote provided). Four OP talked specifically about the programme acting like a ‘catalyst’ that speeded their recovery from depression (quotes provided) | L-M |

Legend 5 *NA= not applicable; NR= not reported; OP= older people (aged 60+ or where the age mean/mode/median is 60+); N= number; Risk of bias: H= high; M= medium; L= low; QUAL= qualitative; MIXED= mixed methods; Adapted from Harden et al.* [62] *and Mays & Pope* [63]
