# Supplementary material for: What is the impact on health and wellbeing of interventions that foster respect and social inclusion in community-residing older adults? A systematic review of quantitative and qualitative studies
Source: Syst Rev. 2018 Jan 30;7:26. doi: 10.1186/s13643-018-0680-2 (PMC5789687; doi:10.1186/s13643-018-0680-2)
Supplement: Supplementary file 6 — Item-level risk of bias (RoB) assessment for quantitative studies using the Liverpool University Quality Assessment Tool (LQAT) (Pope [61]). Item-level risk of bias assessment for quantitative studies. (DOCX 39 kb) [file 13643_2018_680_MOESM6_ESM.docx]

Additional file 6. Item-level Risk of Bias (RoB) assessment for quantitative studies using the Liverpool University Quality Assessment Tool (LQAT) (Pope [61]).

| **First author, year** | **Selection procedures** | **Baseline Assessment** | **Outcome Assessment** | **Analysis/Confounding** | **Contribution for the review*** | **Global assessment RoB** |
| --- | --- | --- | --- | --- | --- | --- |
| **Mentoring interventions** | | | | | | |
| Dickens 2011 [77] | M | W | M | M | S | L-M |
| Ellis 2004 [65] | W | W | M | W | W | H |
| **Intergenerational interventions (including Ellis 2004)** | | | | | | |
| Chung 2009 [68] | W | M | M | M | M | L |
| De Souza 2007 [81] | M | M | M | M | S | L |
| Hernandez 2008 [87] | W | W | M | M | M | L |
| Hong 2010 [82] | M | M | M | M | M | L |
| Fujiwara 2009 [104] | W | W | M | M | M | M |
| Gaggioli 2014 [75] | W | W | M | M | M | M |
| Murayama 2014 [84] | W | W | W | M | M | M |
| Fried 2004 [76] | M | M | W | W | M | M-H |
| Mendis 1993 [103] | M | W | M | M | M | M-H |
| Newman 1995 [105] | W | W | W | W | W | H |
| Ellis 2004 [65] |  |  |  |  |  | H |
| **Dancing interventions** | | | | | | |
| Houston 2015 [70] | W | W | M | W | M | M |
| Hackney 2007 [71] | W | M | M | W | S | M |
| **Music and singing interventions** | | | | | | |
| Coulton 2015 [91] | M | M | M | M | S | L |
| Cohen 2006 [66] | M | W | M | M | M | L |
| Clift 2011 [92] | M | W | M | M | M | L-M |
| Davidson 2014 [94] | W | W | M | W | M | M |
| Creech 2013 [93] | M | W | M | W | M | M |
| Davidson 2011 [69] | W | W | M | W | M | H |
| **Information-communication technology interventions** | | | | | | |
| Slegers 2008 [67] | M | M | W | M | M | L |
| Woodward 2011 [95] | W | M | W | M | M | M |
| Woodward 2012 [78] | W | M | W | M | M | M-H |
| **Art and culture-based interventions (including Cohen 2006)** | | | | | | |
| Phinney 2014 [96] | W | W | S | W | S | L-M |
| Cohen 2006 [66] | M | W | M | M | M | L |
| Camic 2014 [97] | W | W | M | W | M | M |
| Yuen 2011 [98] | W | W | M | W | M | M |
| Vogelpoel 2014 [74] | W | W | M | W | M | M-H |
| **Multi-activity interventions** | | | | | | |
| Saito 2012 [99] | M | M | M | M | M | L-M |
| Gonyea 2013 [107] | W | W | M | M | M | L-M |
| Kocken 1998 [79] | W | W | M | M | M | L-M |
| Greaves 2006 [100] | W | M | M | W | M | M |
| Ruffing-Rahal 1994 [73] | W | W | M | M | M | M |

Legend 6 Each item was rated as S=strong; M=moderate, W=weak. The global descriptive assessment is given based on appraising the items to give a range from lower to higher RoB: H= high; M= medium; L= low.*Contribution for the review refers to how much information answers the review question: What is the empirical evidence on the impact on health and wellbeing of interventions which foster respect and social inclusion in community-residing older adults?
