# Supplementary material for: What is the impact on health and wellbeing of interventions that foster respect and social inclusion in community-residing older adults? A systematic review of quantitative and qualitative studies
Source: Syst Rev. 2018 Jan 30;7:26. doi: 10.1186/s13643-018-0680-2 (PMC5789687; doi:10.1186/s13643-018-0680-2)
Supplement: Supplementary file 7 — Item-level risk of bias (RoB) assessment for qualitative studies using tools adapted from Harden et al. [62] and Mays and Pope [63]. Item-level risk of bias (RoB) assessment for qualitative studies. (DOCX 23 kb) [file 13643_2018_680_MOESM7_ESM.docx]

Additional File 7. Item-level Risk of Bias (RoB) assessment for qualitative studies using tools adapted from Harden et al. [62] and Mays & Pope [63].

| **First author, year** | **Quality of reporting** | | **Methodology** | | | | | | **Use of strategies to increase reliability and validity** | | | **Extent to which findings reflected participant perspectives: validity,**  **trustworthiness** | **Global assessment RoB** |
| --- | --- | --- | --- | --- | --- | --- | --- | --- | --- | --- | --- | --- | --- |
|  | Clear aims | Clear context | Appropriate design | Adequate sampling methods | Generali-zable sample | Data collection methods described | Data analysis methods described | Adequate amount of data presented | Reliable data collection tools | Valid data collection tools | Reliable data analysis methods |  |  |
| **Mentoring interventions** | | | | | | | | | | | | | |
| Ellis 2003 [85] | P | Y | P | P | N | Y | N | P | P | P | NR | P | M-H |
| **Intergenerational interventions (including Ellis 2003)** | | | | | | | | | | | | | |
| Ellis 2003 [85] | P | Y | P | P | N | Y | N | P | P | P | NR | P | M-H |
| De Souza 2003 [86] | Y | Y | P | Y | P | P | N | P | Y | NR | NR | P | L-M |
| Weintraub 2007 [89] | Y | Y | P | P | P | Y | Y | P | P | NR | Y | P | L-M |
| **Dancing interventions** | | | | | | | | | | | | | |
| Houston 2011 [72] | P | Y | N | P | N | P | NR | P | P | NR | Y | P | M-H |
| Houston 2015 [70] | P | Y | P | P | N | P | NR | P | P | NR | Y | P | M-H |
| **Music and singing interventions** | | | | | | | | | | | | | |
| VarVarigou 2012 [101] | Y | Y | P | P | N | P | P | Y | P | P | NR | P | L-M |
| Skingley 2010 [102] | Y | Y | P | P | N | P | P | P | P | NR | NR | P | M |
| **Information-communication technology interventions** | | | | | | | | | | | | | |
| Schlag 2011 [80] | Y | Y | P | Y | N | P | P | P | P | NR | P | Y | M |
| **Art and culture interventions** | | | | | | | | | | | | | |
| Phinney 2014 [96] | P | Y | P | P | P | P | P | P | P | NR | P | P | L-M |
| Yuen 2011 [98] | Y | Y | P | Y | N | Y | Y | P | P | NR | P | P | L-M |
| Vogelpoel 2014 [74] | P | Y | P | P | N | P | NR | P | NR | NR | NR | P | H |
| **Multi-activity interventions** | | | | | | | | | | | | | |
| Buijs 2003 [90] | Y | Y | P | P | N | Y | P | P | P | NR | P | Y | L-M |
| Greaves 2006 [100] | Y | Y | P | P | N | P | P | P | P | NR | Y | Y | L-M |

Legend 7 Each item was rated as Y=yes; N= No; P= partly; NR= not reported. The global descriptive assessment is given based on appraising the items to give a range from lower to higher RoB: H= high; M= medium; L= low.
